# Spatial Epigenetic Control of Mono- and Bistable Gene Expression

**DOI:** 10.1371/journal.pbio.1000332

**Published:** 2010-03-16

**Authors:** János Z. Kelemen, Prasuna Ratna, Simone Scherrer, Attila Becskei

**Affiliations:** Institute of Molecular Life Sciences, University of Zurich, Zurich, Switzerland; Johns Hopkins University, United States of America

## Abstract

Bistability in signaling networks is frequently employed to promote stochastic switch-like transitions between cellular differentiation states. Differentiation can also be triggered by antagonism of activators and repressors mediated by epigenetic processes that constitute regulatory circuits anchored to the chromosome. Their regulatory logic has remained unclear. A reaction–diffusion model reveals that the same reaction mechanism can support both graded monostable and switch-like bistable gene expression, depending on whether recruited repressor proteins generate a single silencing gradient or two interacting gradients that flank a gene. Our experiments confirm that chromosomal recruitment of activator and repressor proteins permits a plastic form of control; the stability of gene expression is determined by the spatial distribution of silencing nucleation sites along the chromosome. The unveiled regulatory principles will help to understand the mechanisms of variegated gene expression, to design synthetic genetic networks that combine transcriptional regulatory motifs with chromatin-based epigenetic effects, and to control cellular differentiation.

## Abstract 2

Changes in the spatial distribution of regulatory protein binding elements relative to gene coding sequences is sufficient to change gene expression patterns from graded to switch-like.

## Author Summary

In the simplest scenario, a gene is expressed when an activator protein binds to its regulatory sequence, and is silenced when the regulatory sequence is bound by a repressor. Many genes are regulated by both activators and repressors, with the response determined by the combined influence of both factors. When the response is monostable graded, expression is finely tuned to a level that reflects the proportion of the bound activator to the bound repressor. Monostable graded systems allow cells to respond precisely to stimuli. If the response is bistable, the response of each cell depends on whether the activator or the repressor wins. Bistable regulation results in the same gene being expressed in some cells and silenced in others, an outcome that promotes cellular differentiation. It remains unclear, however, how different genetic regulatory structures code for monostable graded and bistable responses. We modeled mathematically the behavior of repressors as they bind to and spread their inhibitory effect along genes and found that the spatial distribution of the binding sites determines which response is chosen. If repressors bind both upstream and downstream of the coding sequence, the response is bistable. If they bind only to one side of the coding sequence, the response is monostable. We confirmed our theoretical findings using synthetic genetic constructs in yeast. These findings help to explain how variations in the location of regulatory elements can lead to cellular differentiation and adaption to varying environments.

## Introduction

Graded and switch-like responses reflect fundamental aspects of the functioning of regulatory networks. A graded, monostable response enables the faithful propagation of a signal, and it is often the default response of simple pathways, but regulatory mechanisms can improve the linearity and the dynamic range of the graded response [1],[2]. Conversely, when the signal strength reaches a threshold value, the switch-like response is often manifested in ON and OFF states within a cell population. This binary response can be induced by positive feedback loops capable of generating bistability, but many other mechanisms can support it by rendering the underlying processes more nonlinear and stochastic [3]–[9]. Positive feedback loops in transcriptional or protein kinase networks have been increasingly recognized as a driving force of cellular differentiation [10],[11]. The components of these networks are dissolved in the cytoplasm or nucleoplasm, and typically have a spatially homogeneous distribution.

In contrast, inhomogeneously distributed regulatory components are frequently observed in eukaryotic transcriptional regulation. Binding of eukaryotic transcriptional factors—activators and repressors—to the DNA can lead to recruitment of enzymes and structural proteins of opposing functions, that induce structural changes and covalent modifications of chromatin, exemplified by acetylation and methylation [12],[13]. This leads to a spatially inhomogeneous distribution of regulators along the DNA, constituting the epigenetic code. Activators loosen the chromatin structure. Conversely, the compaction of chromatin and heterochromatin formation are typically induced by repressors or repressor-recruiting DNA sequences that act or interact over long distances, variously termed as *long-range repressors*, *silencing proteins*, and *silencers* in different systems and organisms [14]–[17].

Genes exposed to the antagonism of activators and repressors or silenced chromosomal regions have been frequently observed to display binary response [13],[18]–[21]. Although regulatory principles underlying the graded and binary responses generated by networks with spatially homogeneously distributed components have been increasingly elucidated, the quantitative aspects of the behavior of epigenetic circuits anchored to the chromosome have remained unclear.

We examined whether the spatial distribution of activator and repressor binding sites influences gene expression to become monostable or bistable. We examined long-range interactions between these sites. Since long intervening DNA sequences can receive signals from endogenous cellular pathways, we used heterologous synthetic gene expression systems precluding pleiotropic cellular effects. Synthetic networks have been instrumental in reconstituting nonmonotonous responses and in revealing the basic principles of binary response and bistability in transcriptional regulatory networks based on feedback or competition of activators and repressors [19],[22]–[26]. We identified a concise nonlinear reaction–diffusion equation that explains gene expression of a large number of genetic constructs with different configurations. We found that binary response is not inherent to repressor proteins exhibiting synergy over long distances. Both graded and binary responses can arise depending on the spatial distribution of the binding sites of the repressors along the DNA.

## Results

### Bistable Synergistic Interaction of Silencing Gradients

**Figure 1:**
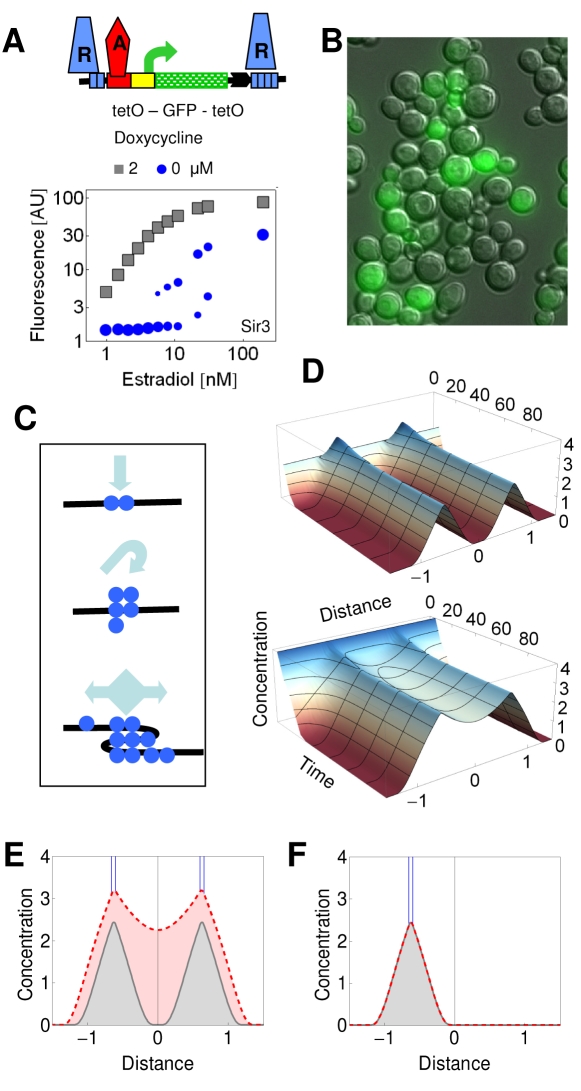
Reaction–diffusion model of bistable repression. (A) In the dual recruitment construct, tetR-Sir3p, denoted as R, binds to the [tetO]_2_ and [tetO]_4_ operators upstream and downstream of the reporter gene, respectively, in the absence of doxycycline. Repression is relieved after addition of *d*  =  2 μM doxycycline, which dissociates tetR from the *tet* operators. Gene expression is activated by the estradiol (*e*)-inducible GEV, denoted as A. The fluorescence value represents the mean of the fitted Gaussian distribution of the cell fluorescence. The area of the circle reflects the proportions of the ON and OFF cells when the distribution was bimodal. (B) Fluorescence and DIC merged images of cells expressing [tetO]_2_-GFP-T-YFP-[tetO]_4_ regulated by tetR-Sir3p. Cells were induced by *e  = * 11 nM in the absence of doxycycline. (C) The steps involved in the reaction–diffusion model (from top to bottom): nucleation, autocatalytic recruitment, and nonlinear diffusion. The S-shaped distortion of the DNA symbolizes the aggregation of the silencing proteins. (D) Evolution of the simulated concentration distributions of silencing proteins along a DNA segment nucleated at two sites. The top and bottom panels show the convergence of the profiles to the steady state representing the low and high silencing states, respectively. The corresponding initial conditions were *c*(*x*, 0)  =  2 and 4. The following parameters were used for Equation 1: *L*  =  5, *K*  =  7, *n*  =  2, *k*_d_  =  1, *b*  =  0.01, and *D*_A_ *= * 1, *s*_h_  =  4, and *s*_w_  =  0.057. The internucleation distance was 1.2 kb. (E) The low (gray continuous line) and the high (red dashed line) concentration profiles represent the long-term solution (200 time units after the initiation) of the model as specified in (D) to reflect the steady state. The blue lines denote the nucleation sites. (F) The two solutions overlap when silencing was nucleated at a single site, calculated as in (E), indicating that the solution is monostable (gray-red dashed line).

Silencing is efficiently induced when multiple silencers interact [14]. To mimic this architecture, we inserted binding sites for the silencing protein Sir3p (in the form of a fusion protein) both downstream and upstream of a gene reporter construct, in the model organism *Saccharomyces cerevisiae*. When recruited to these dual recruitment constructs, Sir3p evoked a variegated GFP expression at intermediate levels of gene activation (*GA*) with a bimodal distribution of cellular fluorescence (Figure 1A and 1B). When *GA* was enhanced, all of the cells switched from the OFF to the ON expression state; so that the ON state was affected only by a residual repression (Figure 1A). Thus, a small change in the input generated a large change in the output. The ON and OFF cell populations represent a simple form of cellular differentiation.

To understand the principles of this form of differentiation, we built a mathematical model based on realistic molecular processes. Due to the complexity and incomplete description of these processes, we sought to identify key mechanisms that can account for bistability in the dual recruitment constructs. The changes in the concentration of the silencing protein at a given point of the space-time, *c*(*x*, *t*), are governed by source *s*(*x*), reaction *r*(*c*), and nonlinear diffusion terms (Figure 1C, Table S1, and Text S1).
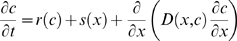
(1)


The nucleation term, *s*(*x*), represents the recruitment of the silencing proteins, and it is a rectangular function. Its width, *s*_w_, is proportional to the number of *tet* operators, while the height of the rectangle, *s*_h_, is proportional to the amount of the silencing proteins recruited to the operators. Thus, *s*_h_ is a function of the doxycycline concentration. The constant nucleation of silencing proteins is necessary for the establishment of steady-state concentration profiles of silencing proteins around the nucleation sites (Figure S1).

Silencing proteins and their cofactors spread along the chromosome, whereby nonspecific protein DNA interactions can facilitate their sliding, a process described by one-dimensional diffusion [18],[27]–[30]. The diffusivity, *D*(*x*, *c*), itself is a variable because the silencing proteins, in particular Sir3, can bridge neighboring DNA segments and condense the chromatin in a concentration-dependent manner, leading the heterochromatin formation [27]. Consequently, the superimposed concentration gradient becomes steeper, accelerating the flux of silencing proteins. Thus, *D*(*x*, *c*) was approximated by *D_A_c*, so that the diffusion term was expressed as 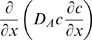. This non-Fickian diffusion term arises in models where diffusional clustering or condensation of particles is described [31],[32].

The reaction term represents an autocatalytic loop based on processes encompassing the cooperative binding of Sir3p and Sir4p, mutual binding of Sir2p, Sir3p, and Sir4p, deacetylation of chromatin by Sir2p creating higher affinity sites for Sir3p and Sir4p, and polymerization of Sir3p proteins [18],[27],[33]–[36].
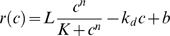



It is assumed that the autocatalytic association of the silencing proteins is superimposed onto a basal, nonspecific association, occurring at a rate of *b*. The former is represented by a Hill function, where *L* stands for the maximal association rate in the limit of *c* → ∞. The dissociation of the silencing proteins is a linear process, and occurs at a rate of *k*_d_.

Initial conditions with uniformly distributed low and high concentrations were used to reflect biochemical fluctuations in the initial accumulation of the silencing proteins (Figure 1D). The simulation of the reaction–diffusion model (Equation 1) revealed that when two silencing nucleation sites were positioned into sufficient proximity, the two initial conditions gave rise to two distinct solutions representing two concentration profiles (Figures 1D and S2). The low-concentration profile was composed of two isolated gradients around the silencing nucleation sites. The high-concentration profile represented a synergistic interaction of the two nucleation sites (Figure 1E).

### Stability Diagram of Gene Expression as a Function of Transcriptional Activation

The coexistence of two concentration profiles for the same parameter values is in accord with the co-occurrence of ON and OFF cells at intermediate *GA* (Figures 1A, 1E, and S3). For a more detailed analysis of bistability, the gene expression has to be calculated from the concentration profiles.

**Figure 2:**
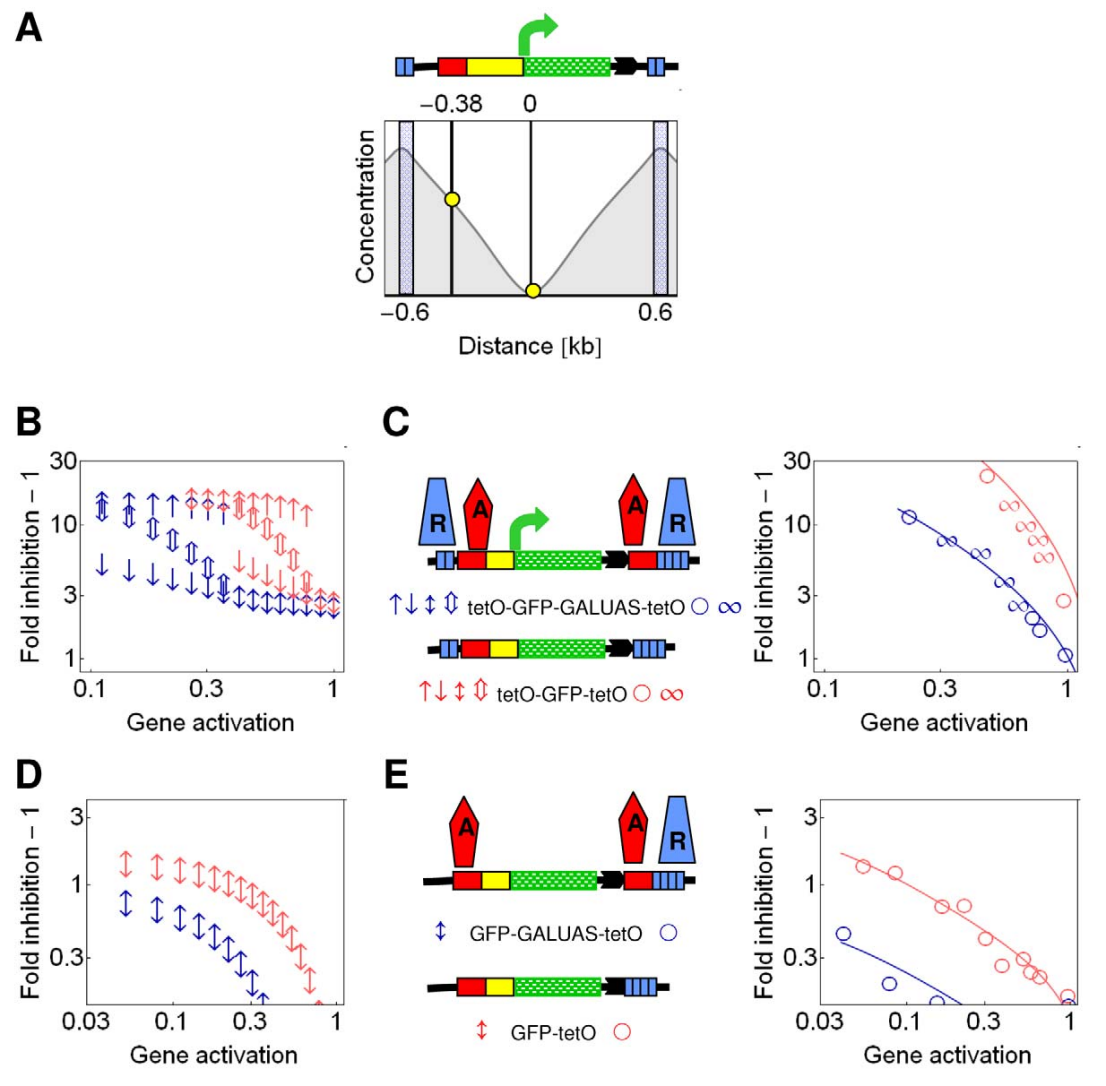
Prediction of gene expression based on the concentration profiles of silencing proteins. The values of the parameters are given in Table S1, unless otherwise indicated. (A) Inhibition of gene expression, expressed as fold inhibition − 1, was calculated from the values of the silencing concentration gradient at the positions *x*_u_  =  −0.38 and *x*_d_  =  0 kb (yellow dots), which span the transcriptional regulatory region of the gene (Equation 2). The upstream point, *x*_u_, corresponds approximately to the region of the activator binding sites while the downstream point, *x*_d_, corresponds to the transcriptional initiation site. These points were chosen as plausible sites of action of silencing proteins. The silencing nucleation sites are positioned at −0.6 and 0.6 kb in the dual nucleation setting. (B) The upwards and downwards arrows represent the solutions initiated with low (*c*(*x*, 0) ≤2) and high (*c*(*x*, 0) ≥4) starting concentrations, for the [O]_2_-Gene-[O]_4_ setting. When the solutions converge, the two arrows merge into an arrow with two arrowheads (monostable region). Double arrows represent weighed mean values of the two solutions to reflect the population average in the bistable region. The red and blue arrows represent solutions with *D_A_*  =  *D*_0_ ˙ *K*_GA_ / (*K*_GA_ + *GA*) and *D_A_*  =  *D*_0_ ˙ *K*_GA_ / 1.36 ˙ (*K*_GA_ + *GA*), respectively. The reduction of diffusivity for the blue arrows reflects the effect of the transcriptional activators bound to the downstream sites that do not contribute to *GA*. (C) *GA* reflects the ratio of expression at the applied estradiol concentration to that at maximal induction (200 nM estradiol), in the absence of repression (*d*  =  2 μM). Fold inhibition − 1 at the applied estradiol concentration reflects the change in gene expression when the repressor binds to the recruitment site (see Materials and Methods). Fold inhibition − 1 was measured for the [tetO]_2_-GFP-[tetO]_4_ (red symbols) and the [tetO]_2_-GFP-GALUAS-[tetO]_4_ (blue symbols) constructs when the fluorescence distributions were unimodal (o) or bimodal (∞). The insertion of the GALUAS did not increase the maximal expression of the construct relative to the control constructs (unpublished data). (D) Calculations performed for the Gene-[O]_4_ setting as in (B). (E) Fold inhibition − 1 was measured for the GFP-[tetO]_4_ (red symbols) and the GFP-GALUAS [tetO]_4_ (blue symbols) constructs, as in (B).

Gene expression is determined jointly by transcriptional activation and silencing. Quantitatively, gene expression is defined as the product of *GA* and fold inhibition due to silencing (see also Materials and Methods). Transcriptional activators not only induce gene expression, but also reduce the spreading of silencing proteins because activators recruit enzymes that relax the structure of chromatin, diminishing the slope of the superimposed concentration gradient [37]. Furthermore, the recruited histone acetyltransferases decrease the number of the available high-affinity binding sites for the silencing proteins [18],[33]. Therefore, the diffusivity was set to be inversely proportional to *GA*, *D_A_* = *D*_0_ ˙ *K*_GA_/(*K*_GA_ + *GA*). Fold inhibition was equated with the concentration of silencing proteins at the gene regulatory region, assuming a linear relation between them. Since repression from the upstream and downstream sites interact multiplicatively [38]:
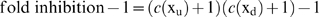
(2)where *x*_u_ and *x*_d_ correspond to the positions −0.38 kb and 0 kb, respectively (Figure 2A).

When *D_A_* was high due to the weak *GA*, simulations initiated with both conditions converged to the synergistically interacting concentration profiles. Correspondingly, gene expression was inhibited strongly. In contrast, the inhibition was weak when *GA* was strong (Figure 2B). At intermediate activation, the strongly and weakly inhibited states co-occurred. In summary, increasing *GA* is accompanied by a transition from the monostable OFF to the monostable ON state through a bistable region, creating a characteristic bifurcation diagram (stability within the mono- and bistable terms refers to the number of steady states) (Figure 2B). The bifurcation diagram was in accordance with the transitions observed for the silenced gene expression as the *GA* was varied experimentally, recapitulating a classical binary response (Figures 1A and 2C).

The model can be validated when further activator binding sites are inserted between the two silencing nucleation sites in a way that they do not contribute to gene expression (Figure 2C). In this case, the model predicted that the bifurcation diagram would not change qualitatively; only the respective stability regions would shift toward the lower *GA* levels since the diffusion of silencing proteins is further diminished (Figure 2B). We tested this prediction by inserting activator binding sites between the terminator of the reporter gene and the *tet* operators, where they do not activate gene expression (Figure 2C). Indeed, bimodal expression was observed for a lower range of *GA* (Figures 2C and S4).

In the above model, the reduction of *D*_A_ between the silencing nucleation sites was spatially uniform. We compared this simple model with a more complex one, in which the reduction of *D*_A_ was more pronounced in the proximity of the activator binding sites. The solutions of the two models were in qualitative agreement (Figure S5).

### Lateral Amplification of Silencing Gradients

**Figure 3:**
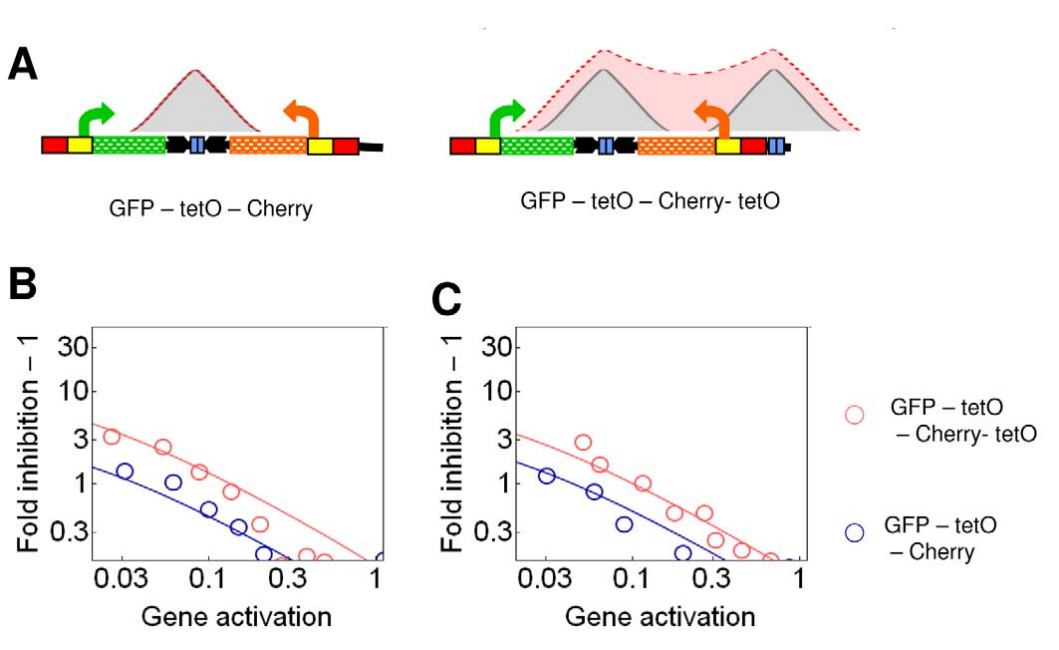
Lateral amplification of silencing gradients. (A) The lateral amplification of silencing gradients can be read out with constructs, in which GFP expression is repressed either by a single downstream cluster of recruitment sites, [tetO]_2_, or by two downstream clusters of recruitment sites separated by a transcription unit, [tetO]_2_-Cherry-[tetO]_2_. (B and C) Fold inhibition − 1 was measured for GFP expression for the GFP-[tetO]_2_ and the GFP [tetO]_2_-Cherry-[tetO]_2_ constructs. The ratio of the inhibition strengths (see Materials and Methods) of the dual recruitment constructs to that of the single recruitment constructs was 3.2 ± 0.31 and 1.77 ± 0.31 for Sir3p (B) and Sum1p (C), respectively.

Whereas the predicted concentration gradient is strongly amplified between the two nucleation sites, a moderate amplification was also predicted for outside of the internucleation segment (Figure 1E). To test this lateral amplification, we compared the inhibition of gene expression when Sir3p was recruited downstream of GFP either to a single site or to two sites separated by a 1-kb-long transcription unit, expressing Cherry (Figure 3A). Indeed, the efficiency of inhibition was stronger by a factor of three for the dual recruitment construct in comparison to the single recruitment construct (Figure 3B), suggesting that the model adequately describes the shape of the gradient. The lateral amplification is predicted to be stronger when *D*_A_ is high (compare Figures 1E, 1F, and S5). The detection of lateral amplification in the convergent transcription constructs (Figure 3A) may have been facilitated by the presence of two terminators separating the GFP and Cherry genes, because silencing, and possibly the spreading of silencing proteins, can be enhanced by transcriptional terminators [38],[39].

### Critical Nucleation Lengths Are Required for Synergistic Bistable Response

**Figure 4:**
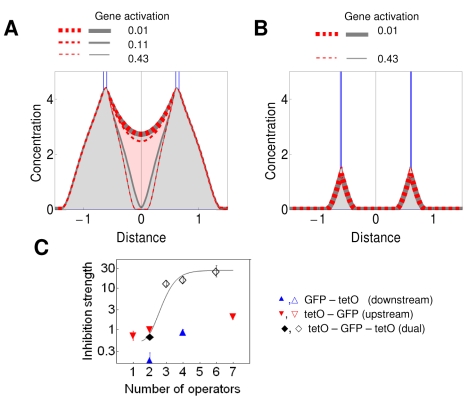
Stability of gene expression and inhibition strength as the function of the number and distribution of nucleation sites. (A and B) Concentration profiles calculated for the [O]_2_-Gene-[O]_2_ (A) and [O]_1_-Gene-[O]_1_ (B) settings. The red dashed and gray continuous lines represent the solutions initiated with the two initial conditions. The two solutions overlap when *GA* is either weak or strong (thin and thick red-gray dashed lines). At intermediate *GA*, two distinct solutions evidenced the bistability (medium red dashed and gray dashed lines) for [O]_2_-Gene-[O]_2_. (C) Inhibition strength at single (upstream or downstream) and dual recruitment constructs. The inhibition strength is the average value for fold inhibition − 1 in the [0.06, 0.6] interval of *GA*. The total number of *tet* operators is indicated for each dual recruitment construct [tetO]_1_-GFP-[tetO]_1_ (*n*  =  2), [tetO]_1_-GFP-[tetO]_2_ (*n*  =  3), [tetO]_2_-GFP-[tetO]_2_ (*n*  =  4), and [tetO]_2_-GFP-[tetO]_4_ (*n*  =  6)_._ Empty symbols stand for constructs that display bimodal gene expression.

Bistable systems can undergo bifurcations with respect to multiple parameters. Therefore, we explored the stability of predicted gene expression as a function of the width of the nucleation sites. The above simulations represented systems with two operators upstream and four operators downstream of the reporter gene (Figure 2B). When the width of the downstream nucleation site was halved, the bistable response persisted: the synergistic monostable, the bistable, and the low monostable concentration profiles alternated as gene expression increased (Figure 4A). Indeed, the experiments utilizing the [tetO]_2_-GFP-[tetO]_2_ construct evidenced the bimodal gene expression at intermediate *GA* and strong average repression (Figure 4C).

When the width of both nucleation segments was halved relative to the previous setting, bistability collapsed, and only the low-concentration profiles were seen over the entire range of *GA* (Figure 4B). In the corresponding experiments, the number of *tet* operators was reduced. The resulting [tetO]_1_-GFP-[tetO]_1_ construct displayed weak silencing and monostable gene expression (Figure 4C), confirming that synergistic interaction of gradients occurs only when the nucleation widths reach a certain threshold.

### The Bistable Response Is Conserved for Repressors Exhibiting Long-Range Synergy

**Figure 5:**
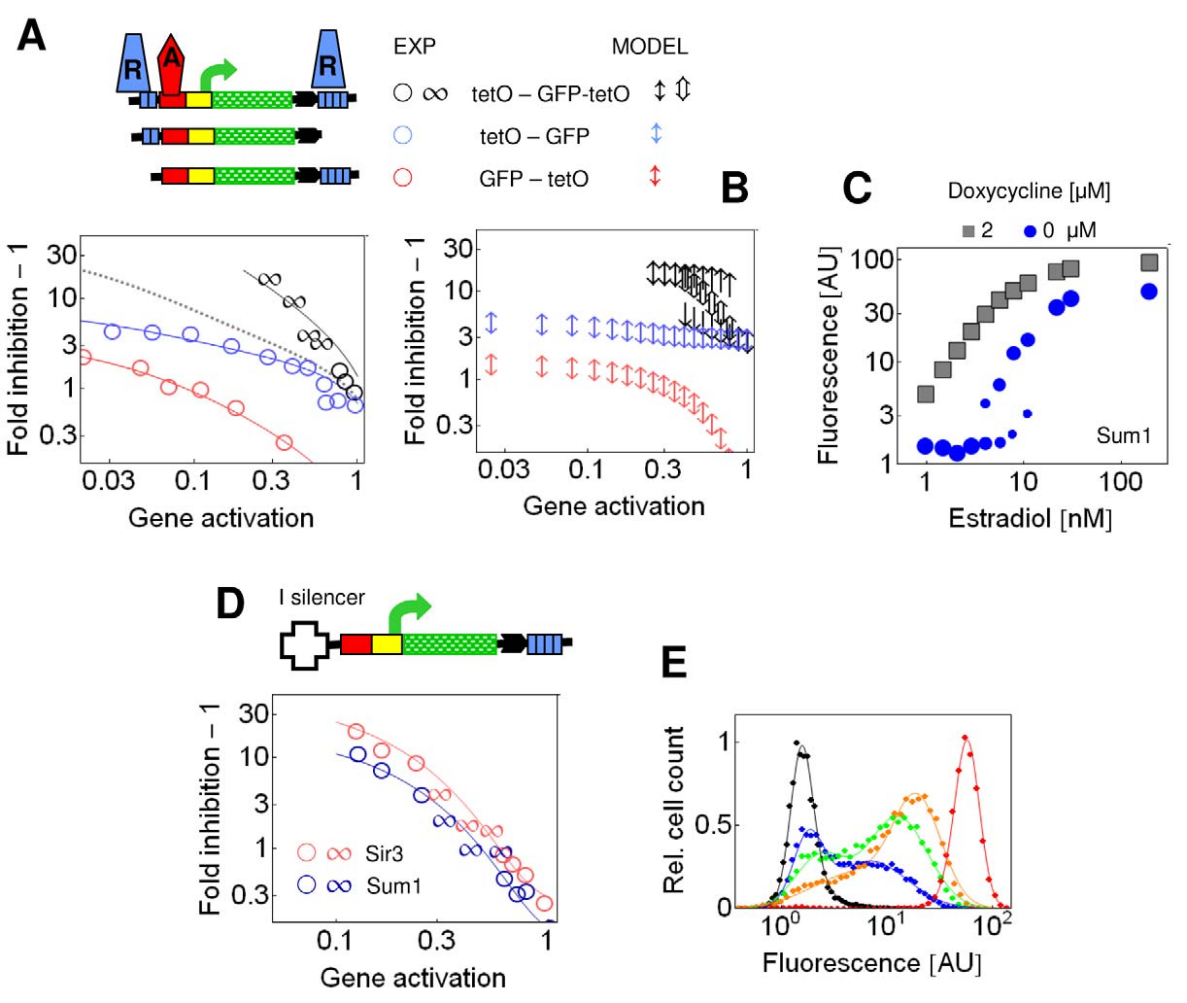
Repression by Sum1p displays long-range synergy and evokes bimodal gene expression in the dual recruitment constructs. The symbols in the fold inhibition plots correspond to those used in Figure 2. (A) tetR-Sum1p was recruited to [tetO]_2_-GFP, GFP-[tetO]_4_, and [tetO]_2_-GFP-[tetO]_4_ constructs. The gray dashed line represents calculated multiplicative interaction of repression from upstream and downstream sites. Fold inhibition − 1 at *GA*  =  0.2 was 4.8 times higher for the dual recruitment construct in comparison to the multiplicative effect, confirming a strong synergy. (B) Calculations performed for the [O]_2_-Gene, Gene-[O]_4_, and [O]_2_-Gene-[O]_4_ setting as in Figure 2B. (C) tetR-Sum1p is recruited to the dual recruitment construct [tetO]_2_-GFP-[tetO]_4_. The fluorescence value represents the mean of the fitted Gaussian distribution of the cell fluorescence. The area of the circle reflects the proportions of the ON and OFF cells when the distribution was bimodal. (D) The inhibition strength at the I silencer-GFP-[tetO]_4_ constructs was 5.91 ± 0.91 and 7.34 ± 2.37 times higher for tetR-Sum1p and -Sir3p, respectively, than that at the parent GFP-[tetO]_4_ constructs. (E) Cellular fluorescence distributions due to the expression of the I silencer-GFP-[tetO]_4_ construct repressed by tetR-Sum1p. Dots are experimental data obtained after adaptive binning, while the lines are fits using two Gaussian distributions. The cells were induced by 1.5, 5.8, 8, 11, and 200 nM estradiol (denoted by black, blue, green, orange, and red colors, respectively), *d*  =  0 μM. AU, arbitrary units.

A model of a biological dynamical system can be corroborated by replacing a network component with a functionally similar component. For this purpose, we tested the Sum1p repressor that binds to the E silencer of the HML heterochromatic locus and contributes to gene silencing [40]. Its cofactor, Hst1p, is a homolog of the silencing protein Sir2p [41]. When Sum1p was recruited as a tetR-Sum1p fusion protein to *tet* operators, it inhibited expression of GFP, independently of whether the *tet* operators were positioned upstream or downstream of the reporter gene (Figure 5A). When bound to both of these sites, Sum1p inhibited gene expression in a strong, synergistic way (Figure 5A). The synergistic interaction over long distance is a phenomenon typical of silencers and repressors acting at heterochromatic loci [14],[42].

At intermediate *GA*, expression of GFP was bimodal (Figure 5C), similar to the observations with Sir3p. The bimodal expression was observed up to 8 h after induction of gene expression (Figure S6). We also examined a well-characterized mutant form of Sum1p, Sum1-1p. This variant was identified in order to efficiently substitute Sir-dependent silencing, and it has a capability to induce pronounced heterochromatin formation [43],[44]. Indeed, Sum1-1p displayed a stronger synergy than Sum1p (Figure S7), and bimodal expression was observed even up to 16 h after induction (Figure S6).

We examined whether Sir3p and Sum1p interacted with the native HML I silencer synergistically. The Sir proteins are recruited to both the E and I silencers, which flank the heterochromatic HML genes, whereas Sum1p is recruited to the E silencer only [40]. The I silencer alone did not have an inhibitory effect on gene expression (Figure S8) [42]. When the reporter gene was flanked by an upstream I silencer and by downstream *tet* operators, both tetR-Sir3p and tetR-Sum1p induced bimodal gene expression at intermediate *GA* (Figures 5D, 5E, and S9).

When the reporter gene was lengthened in the dual recruitment constructs, the synergistic and bistable inhibition of gene expression by Sum1p was abolished (Figure S10). This confirms that in addition to the critical nucleation strength, the two nucleation sites have to be within a critical distance to generate synergistic interaction of the silencing gradients (Figure S5).

In summary, we observed similar responses for four different combinations of silencers and repressor proteins (Figures 2B, 3, and 5), suggesting that they follow the same regulatory principle that associates the synergistic interaction of repressors over large distances with bistability (Figure 5B).

### Synergistic Repressors Generate Monostable Graded Response When Their Binding Sites Are Clustered in a Single Chromosomal Segment

**Figure 6:**
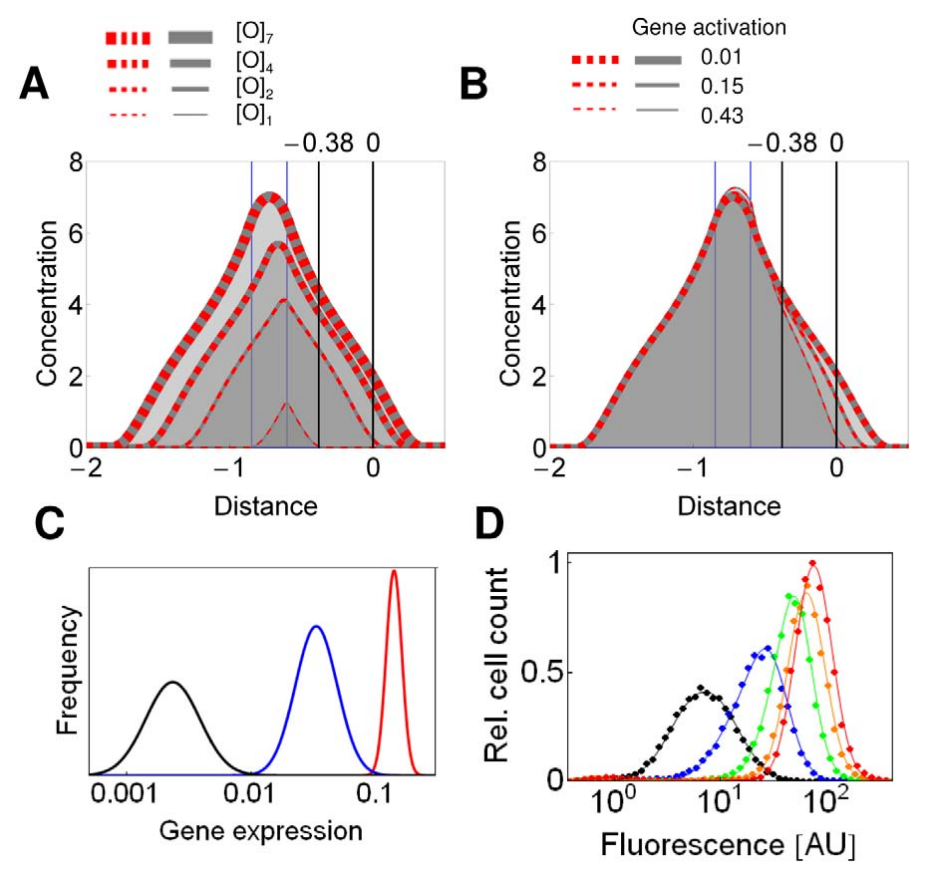
A single cluster of silencing nucleation sites generates a graded, monostable response. (A) The concentration profiles were calculated when *GA* was set to 0.022 and silencing was nucleated at −0.6 kb. The nucleation site comprised one, two, four, and seven operators. The blue lines denote the width of the [O]_7_ nucleation site. The gray continuous and red dashed lines represent the simulated solutions initiated with low and high concentrations, *c*(*x*, 0), respectively. When they overlap, the system is monostable (red-gray dashed lines). (B) The concentration profiles were calculated for [O]_7_ as in (A), but *GA* was varied. (C) Gene expression was calculated from (B) by setting the maximal value of unrepressed gene expression to 1 (see Materials and Methods), so that the black, blue, and red lines correspond to a *GA* of 0.01, 0.15, and 0.43, respectively. A lognormal distribution was assigned to each calculated mean value. (D) Cellular fluorescence distributions due to the expression of [tetO]_7_-GFP, repressed by tetR-Sir3p (YSSD227.4) The cells were induced by 2.9, 5.7, 11, 22, 32, and 200 nM estradiol, in the absence of doxycycline.

Surprisingly, when the silencing proteins were nucleated at a single segment, only one solution emerged using the same parameter values that generated bistability with the dual nucleation setting (Figure 1F and S2). This gradient generated by the single nucleation site was identical with the nonsynergistic solution of the dual nucleation setting (Figure 1E and 1F). Even when the single nucleation segment was broadened, the concentration profiles rose, but they remained monostable over the entire range of *GA* (Figure 6A–6C). Indeed, expression was monostable and responded in a graded way to the binding of Sir3p to upstream regions of promoters containing up to seven operators (Figures 4C and 6D). Monostable graded response was also observed for the entire range of *GA* when tetR-Sum1p and tetR-Sir3p bound to four sites downstream of reporter genes (Figures 2E and 5A). The insertion of activator binding sites in-between the terminator of the reporter gene and downstream operators alleviated the inhibition of gene expression (Figure 2D and 2E), similar to the case for the dual recruitment constructs (Figure 2B and 2C).

**Figure 7:**
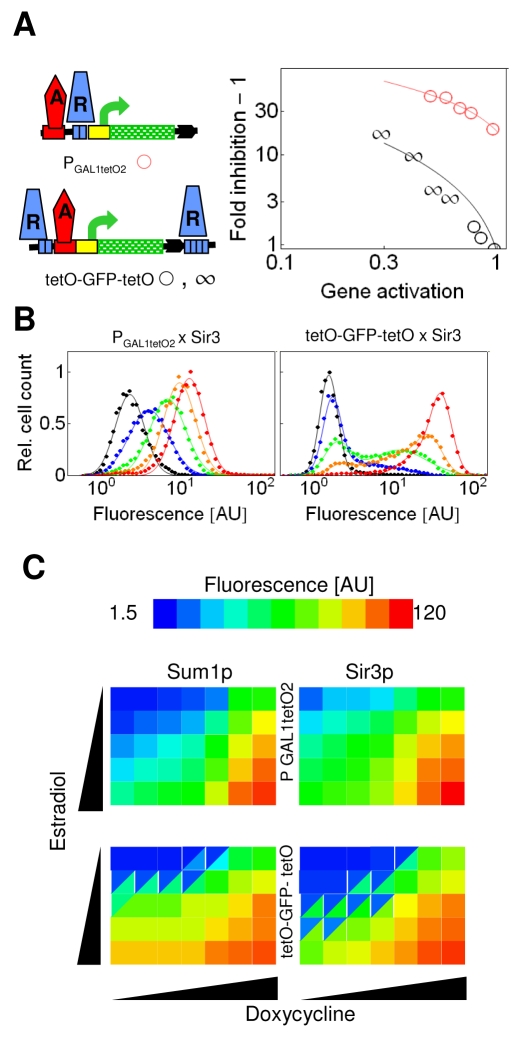
Graded responses can be generated by both Sum1p and Sir3p even when they strongly repress gene expression. (A) P_GAL1tetO2_ corresponds to the *GAL1* promoter, in which the Mig1p binding sites, positioned between the GALUAS and the TATA box, were replaced by *tet* operators. Fold inhibition of gene expression of the respective constructs was obtained for unimodal (o) and bimodal (∞) distributions. (B) Gaussians were fit to fluorescence distributions induced by 3.75, 7.5, 15, 30, and 200 nM estradiol, in the absence of doxycycline. AU, arbitrary units. (C) The means of the fitted Gaussians are color coded. When the distributions were bimodal, the squares were split into two triangles of different colors. The cells were induced by 3.75, 7.5, 15, 30, and 200 nM estradiol and 0, 10, 20, 40, 80,160, and 2,000 nM doxycycline.

None of the above single recruitment constructs with operators clustered to a single chromosomal segment displayed bimodal gene expression. However, they all inhibited gene expression less than the dual recruitment constructs displaying synergistic inhibition of gene expression (Figure 4C). Thus, we hypothesized that bistability was not observed because the inhibition strength did not reach a critical value. In other words, the possibility cannot be excluded that if silencing nucleated at a single cluster inhibited gene expression strongly enough, then the response would be binary. Therefore, we searched for single recruitment constructs with strong inhibitory potential. Fortuitously, when the *tet* operators were inserted between the activator binding sites and the TATA box, a strong inhibition of expression by both Sum1p and Sir3p was observed. In particular, Sum1p inhibited gene expression more strongly when bound to these intercalated operators in comparison to when Sum1p repressed gene expression synergistically in the dual recruitment constructs (Figure 7A).

However, gene expression responded in a graded way over a broad range of activator and repressor binding when Sum1p or Sir3p bound to the intercalated operators (Figure 7B, 7C, and S11). In contrast, the dual recruitment constructs displayed bimodal gene expression when the binding of the activator and repressor was balanced (Figure 7C). The region of bistability was broader for Sir3p in comparison to Sum1p (Figure 7C), in accordance with the stronger synergistic repression and lateral amplification of the gradient by Sir3p (Figure 3B and 3C) [38].

Thus, our experiments confirmed the predictions of the reaction–diffusion model, revealing that the same mechanism can support both graded and binary gene expression depending on the spatial distribution of silencing nucleation sites. Monostable graded expression was characteristic of single nucleation constructs, whereas binary expression was found when two nucleation sites flanked a gene. The OFF and ON cells reflect the effect of the synergistically interacting and isolated silencing gradients, respectively (Figures 1E, 1F, and 4A). Thus, the ON cells are inhibited to a degree comparable to the repression of single nucleation constructs when *GA* is strong (Figure 5A–5C), whereas the OFF cells are inhibited synergistically.

A further exploration of the model revealed a high degree of plasticity of system behavior depending on the parameter values. In particular, the dual nucleation setting generated a graded response when the cooperativity of binding of silencing proteins was reduced (Figure S12). Furthermore, the single nucleation setting displayed bistability when the ratio of the diffusivity to the nucleation width was reduced. In the latter case, however, the silencing proteins did not propagate to long distances due to the low diffusivity, and consequently, they may have no or little impact on gene expression (Figure S13). It remains to be determined whether epigenetic silencing processes exist that assume such parameter values and display behaviors reproducing the above predictions.

## Discussion

Eukaryotic transcriptional *cis* regulation governs developmental and differentiation programs [45]. Long-range interaction between transcription factors makes the deciphering of the logic of this regulation difficult [16],[17],[46]. Whereas long-range interactions can occur even in prokaryotes through looping of the intervening DNA sequences, the long-range effects of eukaryotic activators (enhancers) and repressors (silencers) are often mediated by cofactors that spread along the chromatin, modifying its composition and conformation. Therefore, eukaryotic transcriptional *cis* regulation requires complex spatiotemporal models to understand its logic.

We have devised a concise reaction–diffusion model that captures the important molecular aspects of long-range synergistic repression: autocatalytic recruitment of proteins and their spreading along the DNA that is accompanied by aggregation and condensation of chromatin. We presented a number of experimental tests that confirmed the model predictions. The central result of the model is that the response type depends on the distribution silencing nucleation sites. When two clusters of nucleation sites flank a gene, the system is bistable. For the corresponding genetic constructs, stochastic gene expression with ON and OFF cells was observed. On the other hand, a monostable graded response was generated when silencing was nucleated at a single cluster even if it was relatively long.

Both types of distributions of recruitment clusters for repressors and silencing proteins have been encountered in the genome. An increasing number of promoters have been identified that are dynamically regulated by a single group of binding sites for long-range repressors even within euchromatic regions [41],[47],[48]. In such cases, monostable graded expression is expected to be generated by repressors that follow the regulatory mechanisms we identified.

On the other hand, the synergistic interaction of two or more silencers scattered through telomeric and subtelomeric regions is thought to be required for efficient heterochromatin formation in a broad range of organisms, including yeasts and the mammalian X chromosome [14]. The identification of such silencers is hampered by the fact that in isolation, they lose their silencing capability or may even activate gene expression, so a large number of protosilencers may be hidden in the genome [14]. Genes flanked by two or more silencers are expected to display a stochastic binary expression. Indeed, genes positioned to subtelomeric domains frequently display bimodal and stochastic gene expression in response to environmental stimuli [20],[21],[49]. For example, cell adhesion proteins are localized to subtelomeric domains and are expressed in a variegated way. This phenotypic diversity may enhance the survival and virulence of fungal cells [20],[21]. Conversely, position-effect variegation, a phenomenon characterized by stochastic bimodal expression of a gene positioned to the silenced domains of the chromosome, can arise due to chromosomal aberrations and lead to developmental abnormalities and diseases [50]–[52]. Interaction between multiple silencing gradients can also contribute to correlations in the stochastic fluctuations of expression of genes ordered along the chromosome [53],[54].

Components or mechanisms employed in silencing are often conserved between yeast and higher organisms [33]. Long-range repression and heterochromatin formation can be efficiently reconstituted by tethering the appropriate proteins (or RNA) to the chromosome in different organisms [19],[34],[55],[56]. Therefore, well-defined genetic systems comparable to ours can be employed to examine if the regulatory logic we unveiled is evolutionarily conserved.

**Figure 8:**
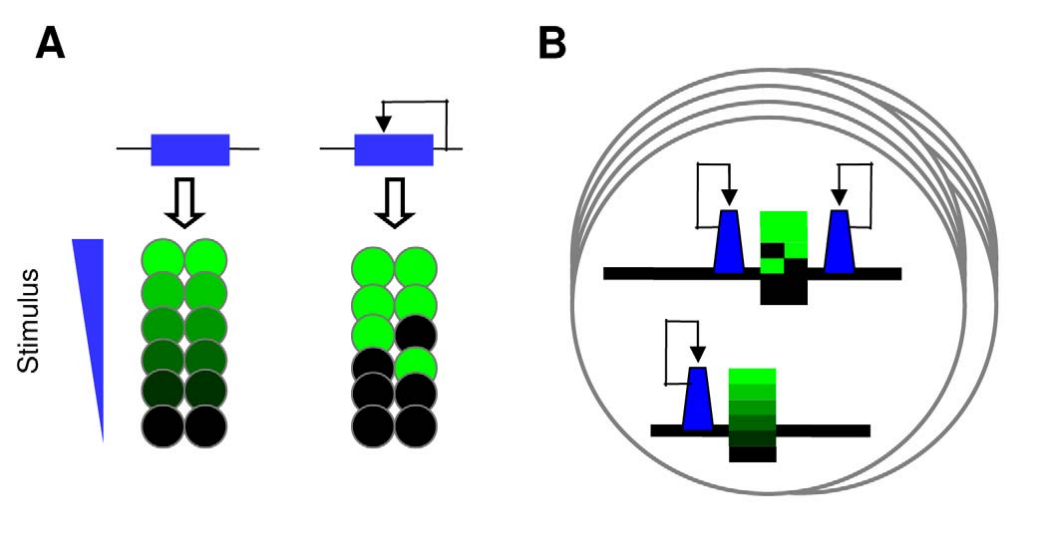
Control modes of dissolved and anchored regulatory circuits. (A) A regulator dissolved in the protoplasm under constitutive or autocatalytic control can trigger either a graded or binary response in a cell population. (B) A regulator anchored to the chromosome can trigger both graded and binary responses at different genes (black-green rectangles) within a single cell.

Our results highlight a difference between signal transducers dissolved in the cell protoplasm and regulatory circuits anchored to the chromosome. Dissolved kinases or transcription factors produce either a monostable or bistable response in a single cell depending on whether they are constitutively regulated or embedded in feedback loops (Figure 8A). In contrast, the same long-range repressor can evoke a monostable graded response at one gene but can induce stochastic transitions between ON and OFF states at another gene (Figure 8B). The outcome is determined by the distribution and density of the recruitment sites of silencing proteins and activators.

The dissolved cellular regulatory networks and the spatially inhomogeneously distributed chromosomal epigenetic circuits will jointly determine gene expression and stability of cellular differentiation states [54],[57]–[59]. Knowing the regulatory principles of the latter will certainly help to decipher their interaction and to understand how they shape cellular functioning.

## Materials and Methods

### Strain Construction and Growth Conditions

The expression of GFP from chromosomally integrated constructs was activated by GEV, an estradiol (*e*)-inducible transcriptional activator, when bound to the GALUAS, and was repressed by tetR fusion proteins (Tables S2 and S3). tetR dissociates from the *tet* operators in the presence of doxycycline (*d*), and repression was relieved at *d* = 2 µM.

GEV is integrated into the genome into the *MRP7* locus; having five copies in the resulting YSSH208. The plasmids containing the tetR-Sir3p and tetR-Sum1p constructs were integrated into the *RET2* locus. The GFP reporter constructs were integrated into the *YFR054c* locus, unless otherwise specified.

Cells containing inducible gene expression constructs were grown for 4 h after induction in minimal media, until a cell density of OD_600_ = 0.4–0.8.

### Analysis of Mean Expression Values

Cellular fluorescence *F_e,d,_* was measured by flow cytometry. Total fluorescence of at least 5,000 cells was measured using flow cytometry. Five to 15% of the total cell population was selected in the forward-scatter versus side-scatter plot to measure GFP fluorescence of cells with similar size.

*GA* is the uninhibited expression at a given estradiol concentration normalized by the maximally induced uninhibited expression (*e* = 0.2 µM, *d* = 2 µM).
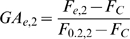



*F_C_* is the background fluorescence of the cells. Fold inhibition is the ratio of the unrepressed expression to the repressed expression (typically at *d* = 0), at a given degree of activation. 
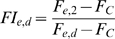



Thus, normalized gene expression is the product of *GA* and fold inhibition at given concentrations of estradiol and doxycycline.

Typically, the OFF cells had fluorescence levels very close to the cellular fluorescence background, which implies that the values of fold inhibition − 1 calculated for the OFF cells after histogram fitting are associated with large measurement errors. For this reason, we calculated fold inhibition − 1 for the entire cell population, which has a higher fluorescence value.

The inhibition strength is the average value for fold inhibition − 1 in the interval *GA* = [0.06, 0.6]. Error bars represent standard deviations calculated from three experiments.

### Histogram Fitting and Bimodality Detection

The logarithmic cellular fluorescence intensities of more than 30,000 cells were extracted from list mode files. The data were subjected to an adaptive binning algorithm [60] to determine the number of bins, and hence, a sampled function of the distribution. A mixture of two Gaussians (Equation 3) was then fitted to each discrete distribution using nonlinear regression.
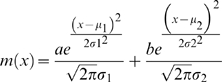
(3)


Finally, the data were transformed from the log space into the linear space.

To systematically detect bimodality in a distribution, we performed the following procedure. The fluorescence distribution was first normalized to a mean of zero, *μ*_M_ = 0, and standard deviation of 1, σ_M_ = 1, and then subjected to binning and regression, as previously described. Subsequently, we considered three metapopulations for the further analysis. The first metapopulation corresponded to the measured events (*M*), with *μ*_M_ = 0 and σ_M_ = 1, since the distribution had been normalized. The population size was normalized to 100. The two remaining metapopulations, denoted *A* and *B*, represented the two fitted Gaussian components (Equation 3) with the mean and variance parameters (*μ_i_*, σ*_i_^2^*) resulting from the nonlinear regression, whereas the respective population sizes *na* and *nb* resulted from the normalization of the coefficients *a* and *b* to the sum of 100, *na* +* nb* = 100. Thus, the sample sizes of *M*, *A*, and *B* were set empirically to correspond to percentages. Next, we performed statistical comparisons between the means of the metapopulations using two-sample *t*-test with unequal variance. When the difference, *μ*_M_ − *μ*_1_ (*μ*_1_ <*μ*_2,_) was significant (*α* = 10^−4)^, the distribution *m*(*x*) was considered bimodal.

## Supporting Information

Figure S1 **Simulated evolution of concentration of silencing proteins in the absence of persistent nucleation,** ***s*** **_h_ = 0.**
An initial pulse was provided in the form of *c*(*x*, 0) = 6 within the segment −0.6< *x* <0.6 kb. *D*_A_ = 0.64. The initiated accumulation of silencing proteins dissipates after around 15 time units, indicating that a constant source of silencing proteins is needed for the maintenance of concentration profiles in the range of parameter values used in our simulations.
(0.12 MB TIF)

Figure S2 **Simulated concentration distribution of silencing proteins along a DNA segment with coarse spatial discretization.**
To account for the compartmental nature of chromatin, we employed the method of finite difference to simulate the model (Equation 1). For the Euler discretization of space and time, the space steps were sized according to the length of the nucleosome (0.16 kb) to ensure the numerical stability of the procedure, the time step was considerably smaller than the space step. The simulation ran to reach 200 time units, similar to the simulations employing the FEM. The concentration profiles are comparable to those in Figure 1E and 1F, using the same kinetic parameters, except for *D*_0_ = 0.5, *s*_h_ = 4; *s*_w_ had to be extended to 0.16 kb, because this is the minimal nucleation width using the coarse spatial discretization. The steady-state concentration profiles were obtained by extending the data points to lines (as with the zero-order hold procedure) to better illustrate the coarseness of the space resolution.
(0.19 MB TIF)

Figure S3 **Parameter dependence of the switch-like transition.**
The surface represents the bistable region, which separates the ON and OFF expression states. *L*, *K*, and *n* were varied in the range [0.5, 10], [0.5, 10], and [1],[3], respectively, with steps of 0.5 units each. The rest of the parameters were kept constant at the same values as used for the dual nucleation model in Figure 1E. Two long-term solutions were calculated, using the low and high initial conditions, to determine the occurrence of bistability. The surface was extrapolated from the points corresponding to parameter triplets (*L*, *K*, *n*) that give rise to bistability. Note, that for *n* = 1 (lack of cooperativity), bistability did not occur.
(0.24 MB TIF)

Figure S4 **Cellular fluorescence distributions due to the expression of the [tetO]_2_-GFP-GALUAS-[tetO]_4_ construct repressed by tetR-Sir3.**
The cells (PRY524.1) were induced by 2.1, 4.1, 8, 16, and 200 nM estradiol in the absence of doxycycline.
(0.19 MB TIF)

Figure S5 **Comparison of the concentration profiles with uniform and nonuniform diffusivities within the [O]_2_-Gene-[O]_4_ setting.**
*GA* reduces the spreading of the silencing proteins, which can be mediated by histone acetylation, and by the activator-induced transcription that disrupts heterochromatin. The former process is expected to reduce diffusivity around the activator binding sites, whereas the latter reduces diffusivity along the entire gene. In the main simulations, the diffusion coefficient was reduced uniformly in the segment flanked by the nucleation sites to imitate reduction of diffusivity along the entire gene (see also [A, C, and E]). For comparison, we simulated concentration profiles when the diffusivity was reduced nonuniformly, around the activator binding sites (B, D, and F). The results are comparable using the two approaches.
(A) *D*_A_ was reduced uniformly as *GA* was increased in-between the nucleation sites, whereas outside of this region, *D*_0_ = 0.64. Curves represent the functions *D*_A_ = 0.52, 0.36, and 0.24.
(B) The nonuniform distribution is given by *D*_A_(*x*) = *D*_0_•(1+*f*σΝ(μ, σ^2^))^−1^ where Ν (μ,σ^2^) denotes the Gaussian distribution with mean μ and variance σ^2^. μ was set to −0.38 kb, which corresponds to the activator binding site, while σ equals the internucleation distance divided by four. *D*_0_ = 0.64. *GA* was increased by setting *f* to 1.5, 6, and 12.
(C and D) The red dashed and gray continuous lines represent the solutions initiated with low and high starting concentrations. The internucleation distance was 1.2 kb.
(E and F) Simulations as performed in (C) and (D), but the internucleation distance was increased to 1.5 kb. Consequently, the synergistic interaction between the two gradients was abolished.
(0.55 MB TIF)

Figure S6 **Long-term changes in the cellular fluorescence distributions due to the expression of the [tetO]_2_-GFP-[tetO]_4_ construct repressed by Sum1p or Sum1-1p.**
The cells were induced by 0, 8, 11.3, 22, and 200 nM estradiol (denoted by black, blue, green, orange, and red colors, respectively), in the absence of doxycycline. Cells were grown exponentially for the period (8 h or 16 h) indicated. Bimodal expression can be seen 16 h after induction by 11.3 nM estradiol due to silencing by Sum1-1p.
(0.45 MB TIF)

Figure S7 **Synergy of repression by Sum1-1p.**
Sum1-1p is the T988I mutant form of Sum1p. tetR-Sum1-1p was recruited to [tetO]_2_-GFP (DHS43), GFP-[tetO]_4_ (DHS44), and [tetO]_2_-GFP-[tetO]_4_ (DHS45) constructs. The gray dashed line represents calculated multiplicative interaction of repression from upstream and downstream sites. Fold inhibition − 1 at *GA* = 0.2 was 13.1 times higher for the dual recruitment construct in comparison to the multiplicative effect, indicating a very strong synergy (see also Figure 5A).
(0.16 MB TIF)

Figure S8 **The I silencer alone does not repress the reporter gene.**
The expression induced by GEV at the I silencer-GFP-[tetO]_4_ construct (PRY544.1, −545.1) was not lower than that at the GFP-tetO4 construct (YJK15), in nonrepressive conditions (tetR-Sum1p and tetR-Sir3p do not repress expression in the presence of 2 µM doxycycline). Thus, the I silencer alone does not repress the reporter gene; it has rather a weak activatory potential.
(0.23 MB TIF)

Figure S9 **Cellular fluorescence distributions due to the expression of the I-silencer-GFP-tetO construct repressed by tetR-Sir3.**
The cells (PRY544.1) were induced by 1.5, 5.8, 8, 11, and 200 nM estradiol, in the absence of doxycycline.
(0.19 MB TIF)

Figure S10 **Collapse of bimodal expression as the distance between the recruitment sites for tetR-Sum1 is increased.**
(A) Sum1p was recruited to the dual recruitment constructs enclosing reporter genes of varying lengths (GFP, [GFP]_2_, GFP-T-YFP, GFP-T-lacZ integrated within the respective strains: YJKD-16, −3.4, −3.5, −3.6). The relative inhibition denotes the inhibition strength (see Materials and Methods) of the dual recruitment constructs normalized using the [tetO]_2_-GFP construct. The inhibition strength is the average value of the fold inhibition − 1 interpolated on the interval *GA* = [0.06, 0.6]. Error bars represent standard deviations calculated from three experiments.
(B and C) Cellular fluorescence distributions due to the expression of the [tetO]_2_-[GFP]_2_-[tetO]_4_ (B) and [tetO]_2_-GFP-T-lacZ-[tetO]_4_ (C) constructs repressed by Sum1p. The cells were induced by 0, 4.1, 5.8, 16, and 200 nM estradiol, in the absence of doxycycline. No bimodal response was detected for the [tetO]_2_-GFP-T-lacZ-[tetO]_4_ construct.
(0.36 MB TIF)

Figure S11 **Cellular fluorescence distributions when expression is repressed by Sum1p.**
The cells (YJKD21.2.2 and YJK16) were induced by 3.75, 7.5, 15, 30, and 200 nM estradiol, in the absence of doxycycline.
(0.24 MB TIF)

Figure S12 **Monostable concentration profiles arise when cooperativity in the positive feedback loop is small.**
The Hill coefficient was reduced from 2 to *n* = 1.5. The following parameters were used for the simulations: *s*_h_ = 6, *L* = 5, *K* = 5, *b* = 0.01, and *k_d_* = 1. The internucleation distance was 1.2 kb for the [O]_2_-Gene-[O]_2_ setting.
(A) The red dashed and gray continuous lines represent the solutions initiated with low and high starting concentrations. The blue lines delimit the nucleation sites. When the two concentration profiles overlap red–gray dashed lines are visible. Monostable concentration profiles were obtained even at intermediate *GA*.
(B) Inhibition of gene expression, expressed as fold inhibition − 1, was calculated from the values of the silencing concentration gradients. Even though there is no bistability at intermediate *GA*, a sigmoidal change in fold inhibition can be seen in this range.
(0.25 MB TIF)

Figure S13 **Bistable concentration profiles are confined to the proximity of the nucleating segment when diffusivity is low relative to the nucleation width.**
The following parameters were used for the simulations: *s*_h_ = 0.3, *L* = 5, *K* = 7, *b* = 0.01, and *k*_d_ = 1 for a [O]_20_-Gene setting. *D*_A_ was set to the indicated values uniformly between the boundaries of the simulation. The blue lines delimit the nucleation segment, *s*_w_ = 0.741 kb. The widening of the nucleation segment and reduction of the diffusivity renders the spatial aspect of the reaction–diffusion system less pronounced. Consequently, the behavior of the systems approximates that of a simple (nonspatial) positive feedback loop that generates bistability. The yellow dots denote the concentrations at −0.38 and 0 kb, which determine the level of *GA*.
(A and B) The red dashed and gray continuous lines represent the solutions initiated with low and high starting concentrations with *D*_A_ = 0.2 (A) and 0.6 (B). Bistable solution is obtained for lower diffusivity, *D*_A_ = 0.2. It is evident that the silencing proteins do not propagate to long distances relative to the width of the nucleation segment and the concentrations of the silencing proteins at the gene regulatory region (yellow dots) are low even for the high-concentration profile. Thus, they have an effect on gene expression only in the vicinity of the nucleating segment.
(C) The magnified version of the low-concentration profiles is displayed for *D*_A_ = 0.2 (thin line) and 0.6 (thick line). It is evident that the concentration profile obtained for the lower diffusivity is more square-like.
(0.29 MB TIF)

Table S1 **Constants used in the equations.**
(0.04 MB DOC)

Table S2 **Strains.**
(0.06 MB DOC)

Table S3 **Plasmids.**
(0.05 MB DOC)

Text S1 **Supporting text and references.**
(0.05 MB DOC)

## Abbreviations

*GA*: gene activation
