# Supplementary figures and images for: Spatial Epigenetic Control of Mono- and Bistable Gene Expression
Source: PLoS Biol. 2010 Mar 16;8(3):e1000332. doi: 10.1371/journal.pbio.1000332 (PMC2838748; doi:10.1371/journal.pbio.1000332)

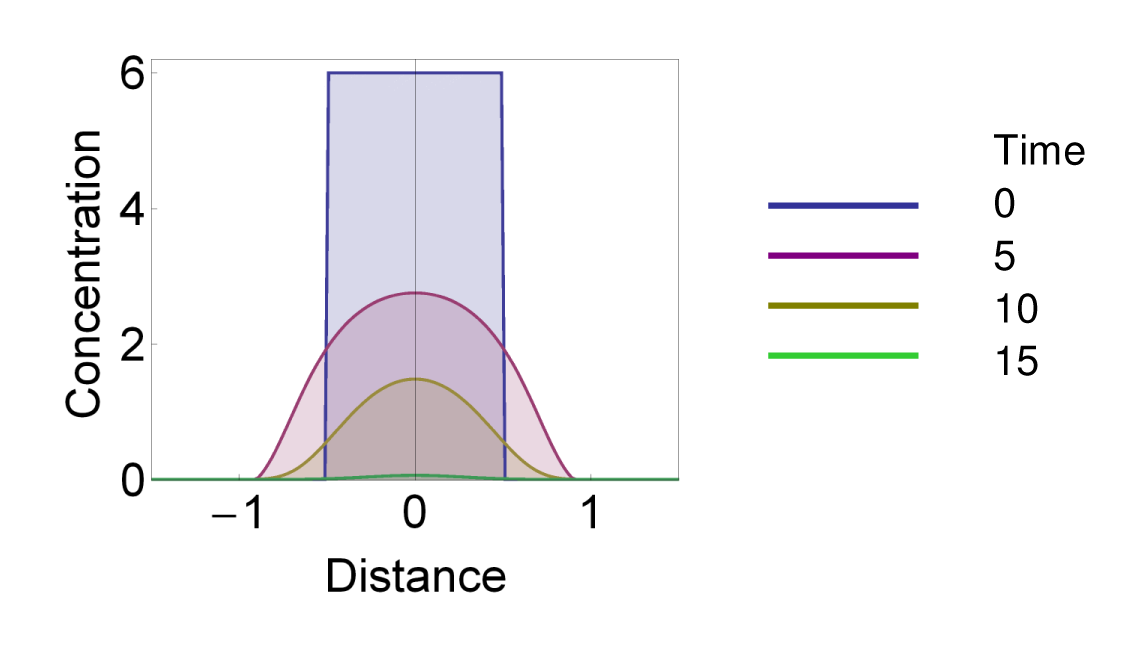

Supplement: Figure S1 — Simulated evolution of concentration of silencing proteins in the absence of persistent nucleation, s h = 0. An initial pulse was provided in the form of c(x, 0) = 6 within the segment −0.6< x <0.6 kb. D A = 0.64. The initiated accumulation of silencing proteins dissipates after around 15 time units, indicating that a constant source of silencing proteins is needed for the maintenance of concentration profiles in the range of parameter values used in our simulations. (0.12 MB TIF) [file pbio.1000332.s001.tif]

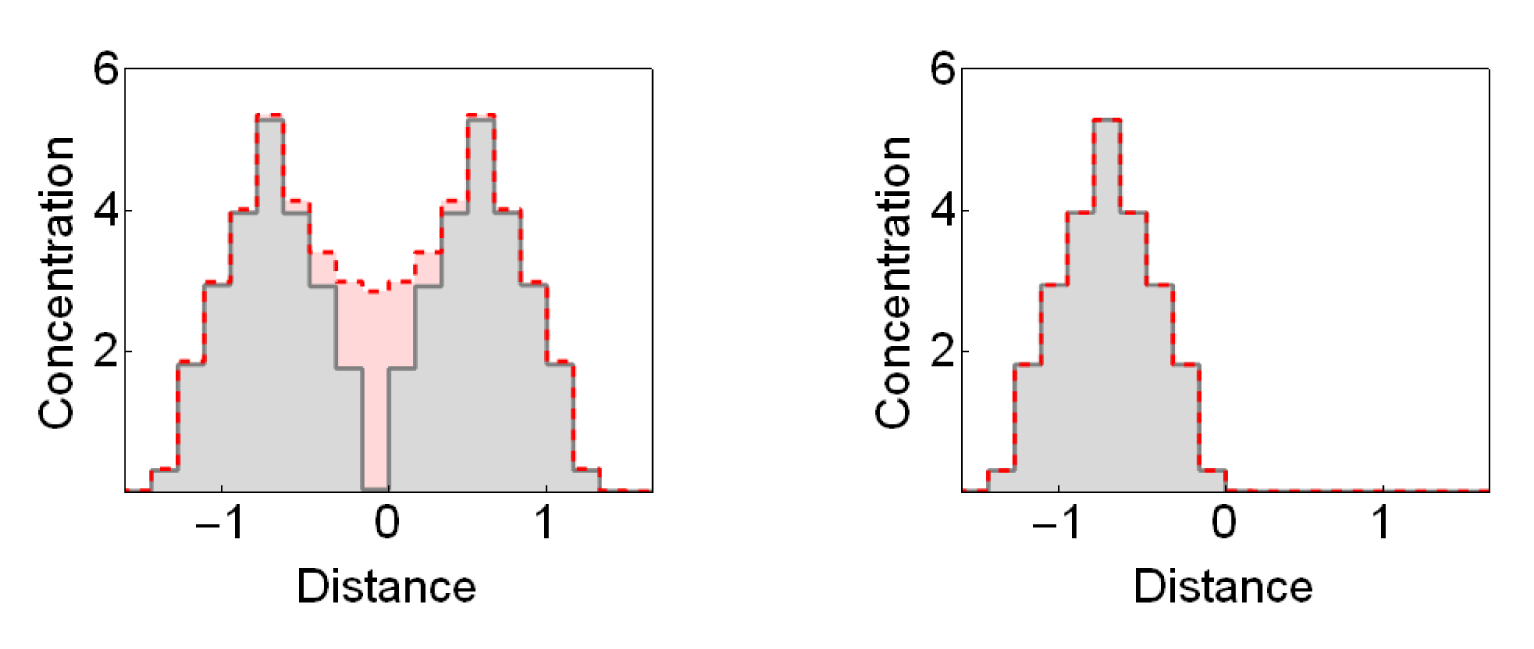

Supplement: Figure S2 — Simulated concentration distribution of silencing proteins along a DNA segment with coarse spatial discretization. To account for the compartmental nature of chromatin, we employed the method of finite difference to simulate the model (Equation 1). For the Euler discretization of space and time, the space steps were sized according to the length of the nucleosome (0.16 kb) to ensure the numerical stability of the procedure, the time step was considerably smaller than the space step. The simulation ran to reach 200 time units, similar to the simulations employing the FEM. The concentration profiles are comparable to those in Figure 1E and 1F, using the same kinetic parameters, except for D 0 = 0.5, s h = 4; s w had to be extended to 0.16 kb, because this is the minimal nucleation width using the coarse spatial discretization. The steady-state concentration profiles were obtained by extending the data points to lines (as with the zero-order hold procedure) to better illustrate the coarseness of the space resolution. (0.19 MB TIF) [file pbio.1000332.s002.tif]

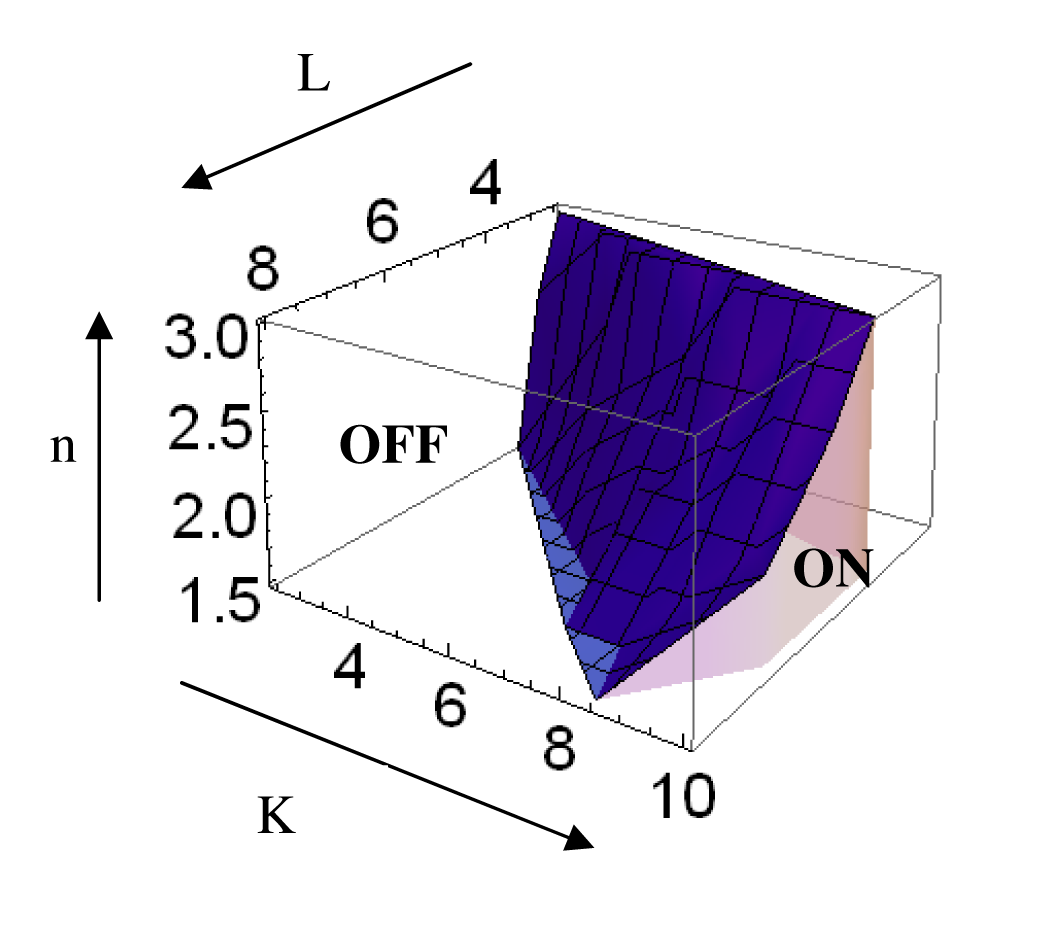

Supplement: Figure S3 — Parameter dependence of the switch-like transition. The surface represents the bistable region, which separates the ON and OFF expression states. L, K, and n were varied in the range [0.5, 10], [0.5, 10], and [1],[3], respectively, with steps of 0.5 units each. The rest of the parameters were kept constant at the same values as used for the dual nucleation model in Figure 1E. Two long-term solutions were calculated, using the low and high initial conditions, to determine the occurrence of bistability. The surface was extrapolated from the points corresponding to parameter triplets (L, K, n) that give rise to bistability. Note, that for n = 1 (lack of cooperativity), bistability did not occur. (0.24 MB TIF) [file pbio.1000332.s003.tif]

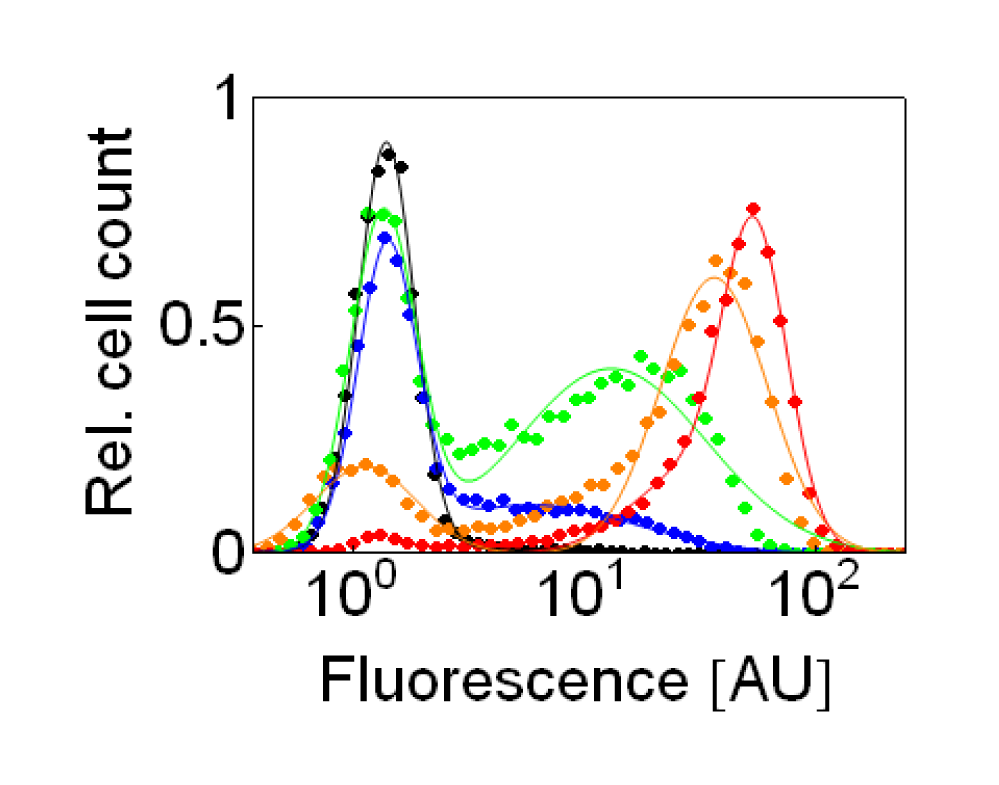

Supplement: Figure S4 — Cellular fluorescence distributions due to the expression of the [tetO]2-GFP-GALUAS-[tetO]4 construct repressed by tetR-Sir3. The cells (PRY524.1) were induced by 2.1, 4.1, 8, 16, and 200 nM estradiol in the absence of doxycycline. (0.19 MB TIF) [file pbio.1000332.s004.tif]

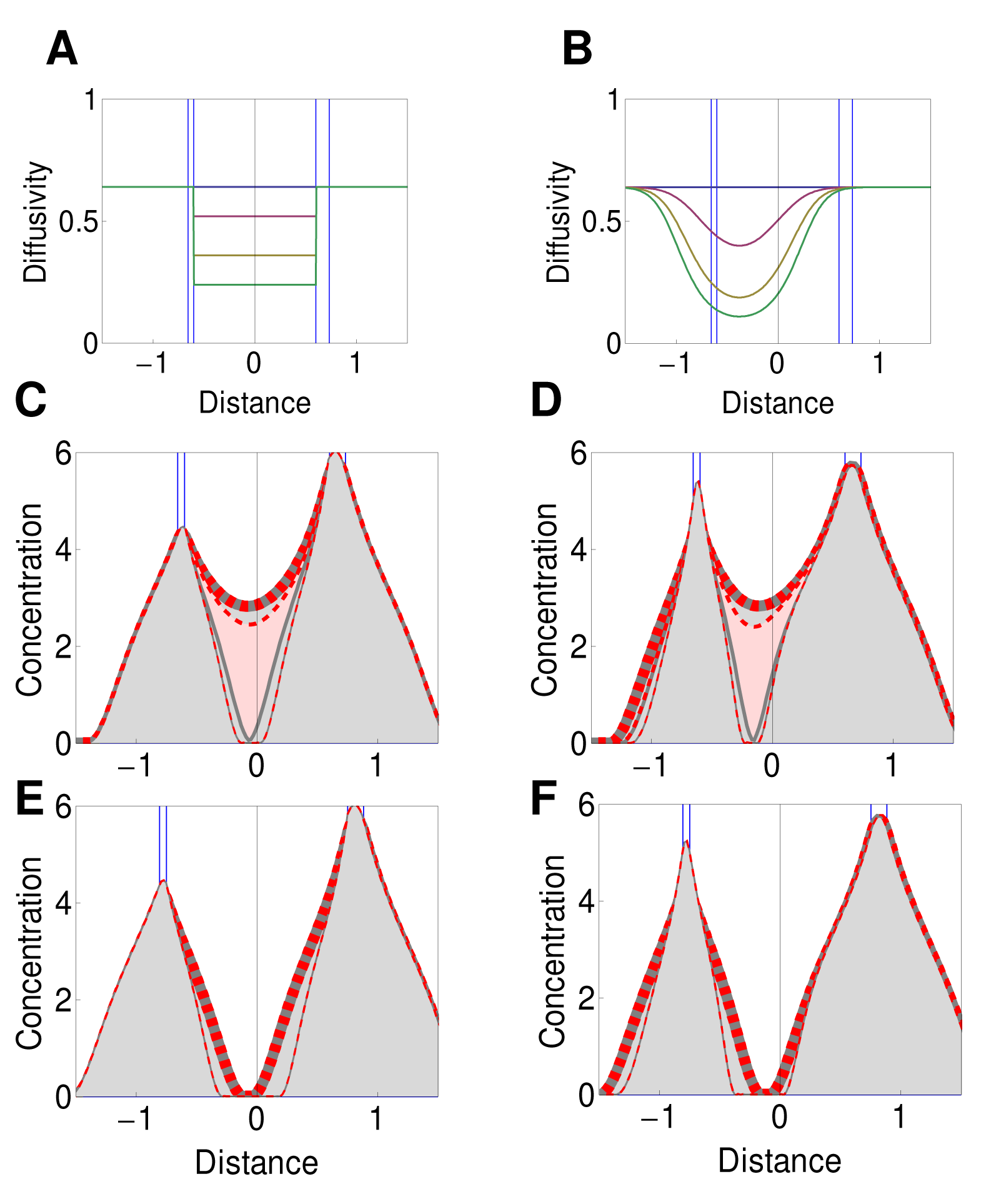

Supplement: Figure S5 — Comparison of the concentration profiles with uniform and nonuniform diffusivities within the [O]2-Gene-[O]4 setting. GA reduces the spreading of the silencing proteins, which can be mediated by histone acetylation, and by the activator-induced transcription that disrupts heterochromatin. The former process is expected to reduce diffusivity around the activator binding sites, whereas the latter reduces diffusivity along the entire gene. In the main simulations, the diffusion coefficient was reduced uniformly in the segment flanked by the nucleation sites to imitate reduction of diffusivity along the entire gene (see also [A, C, and E]). For comparison, we simulated concentration profiles when the diffusivity was reduced nonuniformly, around the activator binding sites (B, D, and F). The results are comparable using the two approaches. (A) D A was reduced uniformly as GA was increased in-between the nucleation sites, whereas outside of this region, D 0 = 0.64. Curves represent the functions D A = 0.52, 0.36, and 0.24. (B) The nonuniform distribution is given by D A(x) = D 0•(1+fσΝ(μ, σ2))−1 where Ν (μ,σ2) denotes the Gaussian distribution with mean μ and variance σ2. μ was set to −0.38 kb, which corresponds to the activator binding site, while σ equals the internucleation distance divided by four. D 0 = 0.64. GA was increased by setting f to 1.5, 6, and 12. (C and D) The red dashed and gray continuous lines represent the solutions initiated with low and high starting concentrations. The internucleation distance was 1.2 kb. (E and F) Simulations as performed in (C) and (D), but the internucleation distance was increased to 1.5 kb. Consequently, the synergistic interaction between the two gradients was abolished. (0.55 MB TIF) [file pbio.1000332.s005.tif]

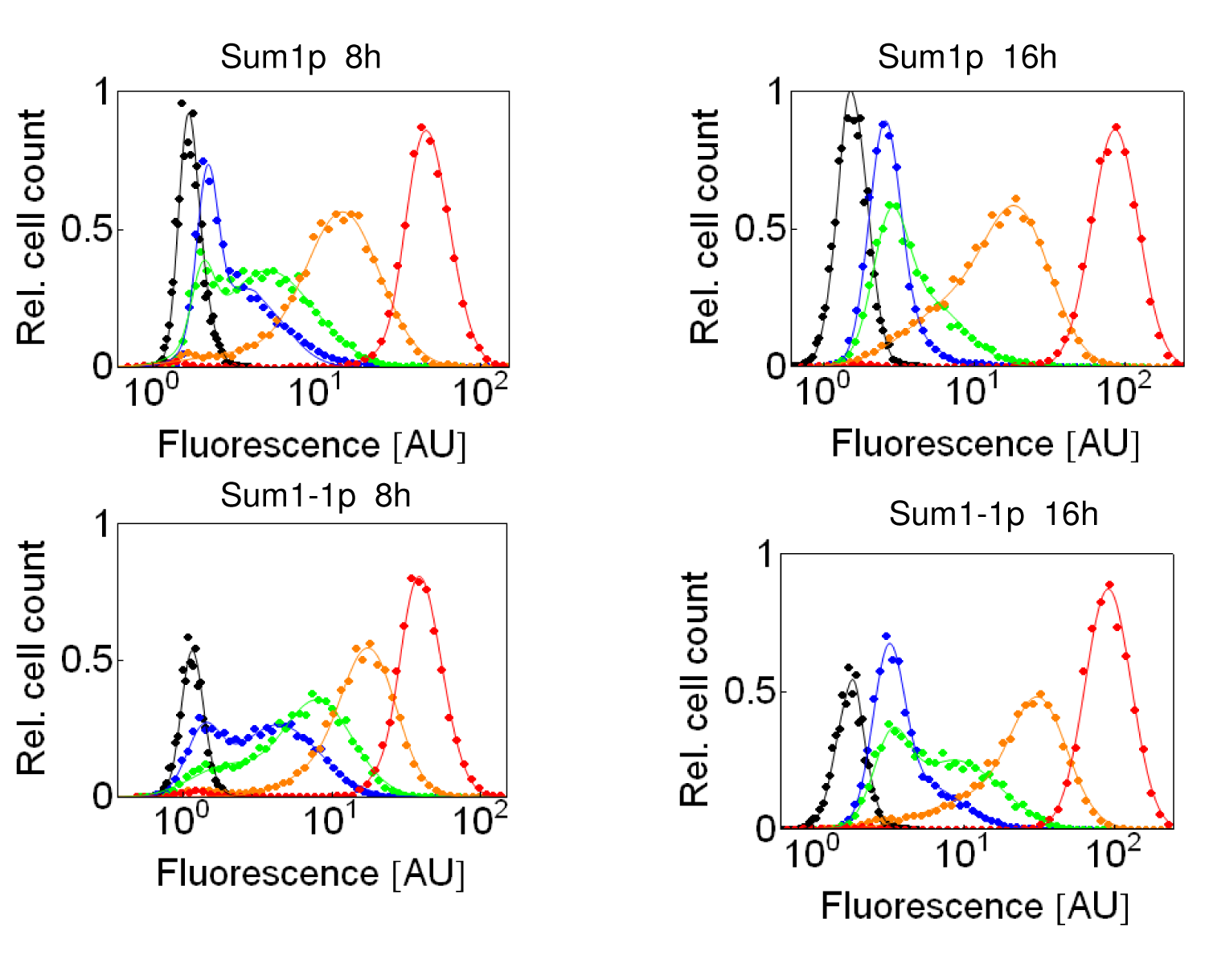

Supplement: Figure S6 — Long-term changes in the cellular fluorescence distributions due to the expression of the [tetO]2-GFP-[tetO]4 construct repressed by Sum1p or Sum1-1p. The cells were induced by 0, 8, 11.3, 22, and 200 nM estradiol (denoted by black, blue, green, orange, and red colors, respectively), in the absence of doxycycline. Cells were grown exponentially for the period (8 h or 16 h) indicated. Bimodal expression can be seen 16 h after induction by 11.3 nM estradiol due to silencing by Sum1-1p. (0.45 MB TIF) [file pbio.1000332.s006.tif]

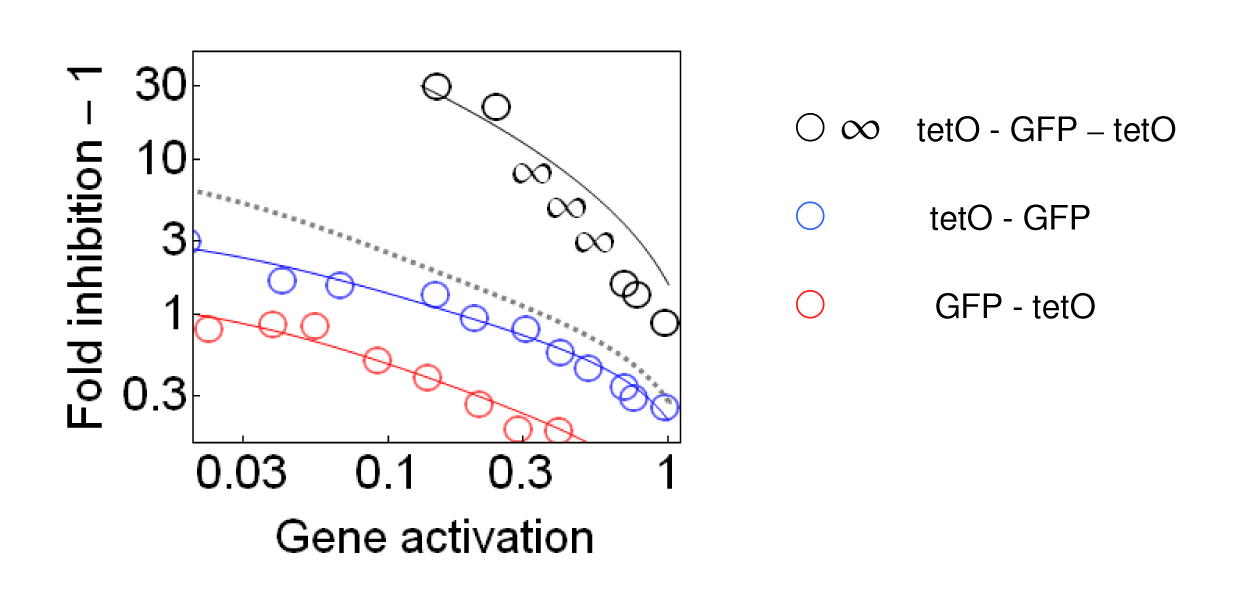

Supplement: Figure S7 — Synergy of repression by Sum1-1p. Sum1-1p is the T988I mutant form of Sum1p. tetR-Sum1-1p was recruited to [tetO]2-GFP (DHS43), GFP-[tetO]4 (DHS44), and [tetO]2-GFP-[tetO]4 (DHS45) constructs. The gray dashed line represents calculated multiplicative interaction of repression from upstream and downstream sites. Fold inhibition − 1 at GA = 0.2 was 13.1 times higher for the dual recruitment construct in comparison to the multiplicative effect, indicating a very strong synergy (see also Figure 5A). (0.16 MB TIF) [file pbio.1000332.s007.tif]

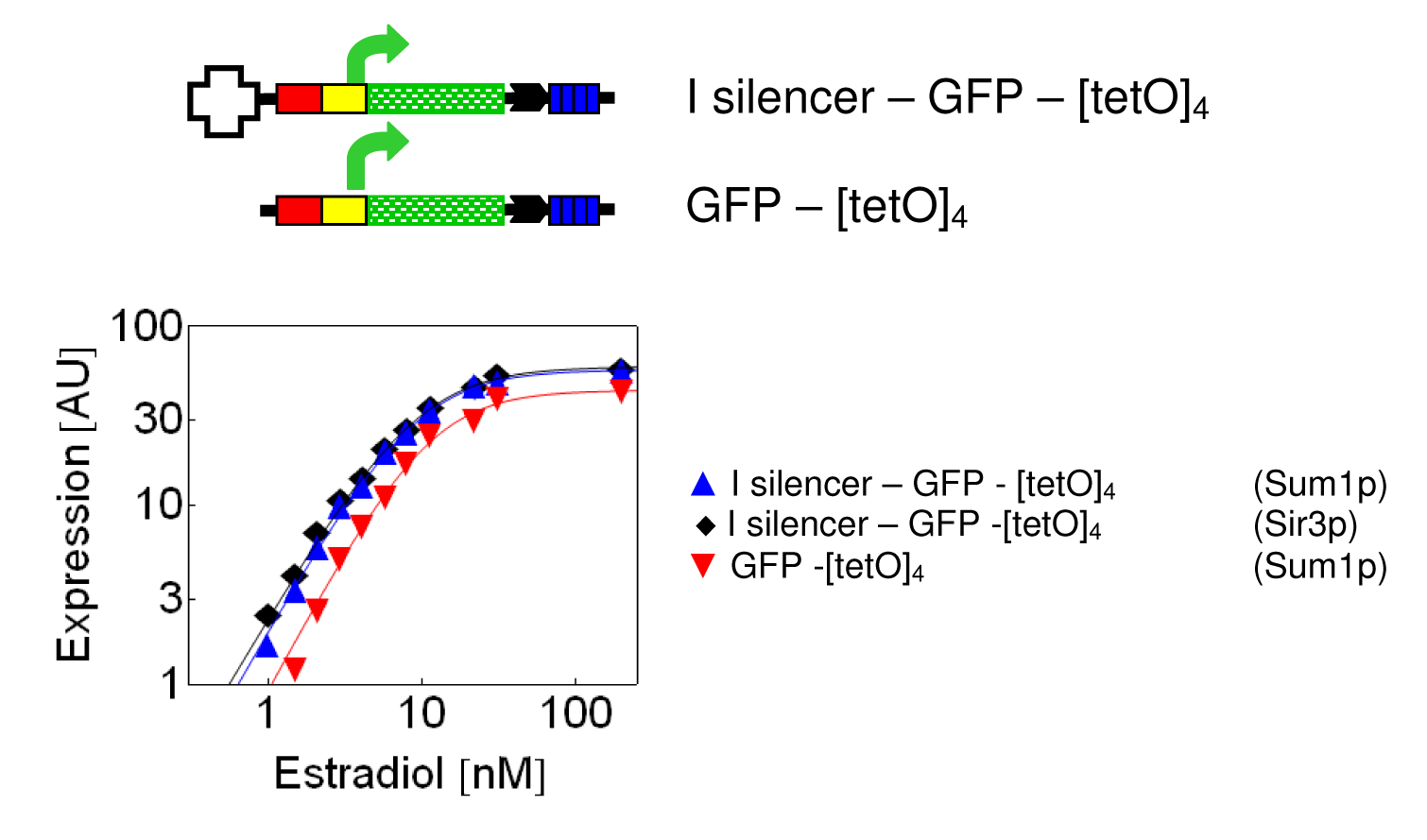

Supplement: Figure S8 — The I silencer alone does not repress the reporter gene. The expression induced by GEV at the I silencer-GFP-[tetO]4 construct (PRY544.1, −545.1) was not lower than that at the GFP-tetO4 construct (YJK15), in nonrepressive conditions (tetR-Sum1p and tetR-Sir3p do not repress expression in the presence of 2 µM doxycycline). Thus, the I silencer alone does not repress the reporter gene; it has rather a weak activatory potential. (0.23 MB TIF) [file pbio.1000332.s008.tif]

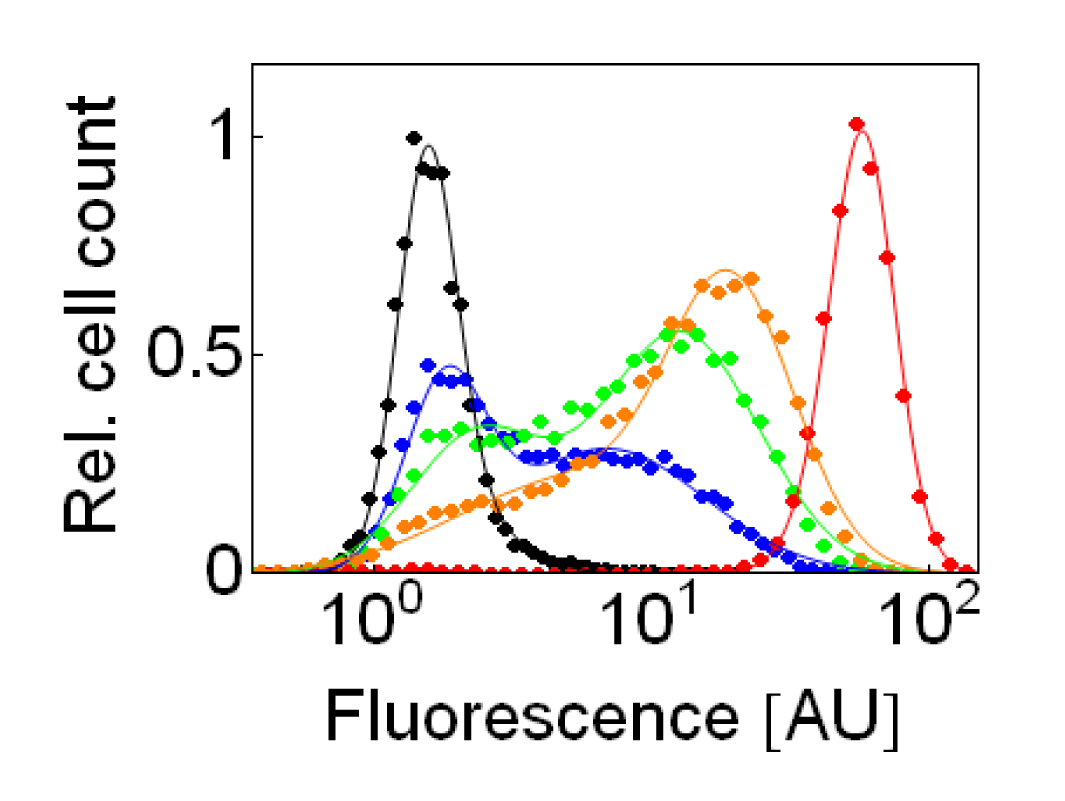

Supplement: Figure S9 — Cellular fluorescence distributions due to the expression of the I-silencer-GFP-tetO construct repressed by tetR-Sir3. The cells (PRY544.1) were induced by 1.5, 5.8, 8, 11, and 200 nM estradiol, in the absence of doxycycline. (0.19 MB TIF) [file pbio.1000332.s009.tif]

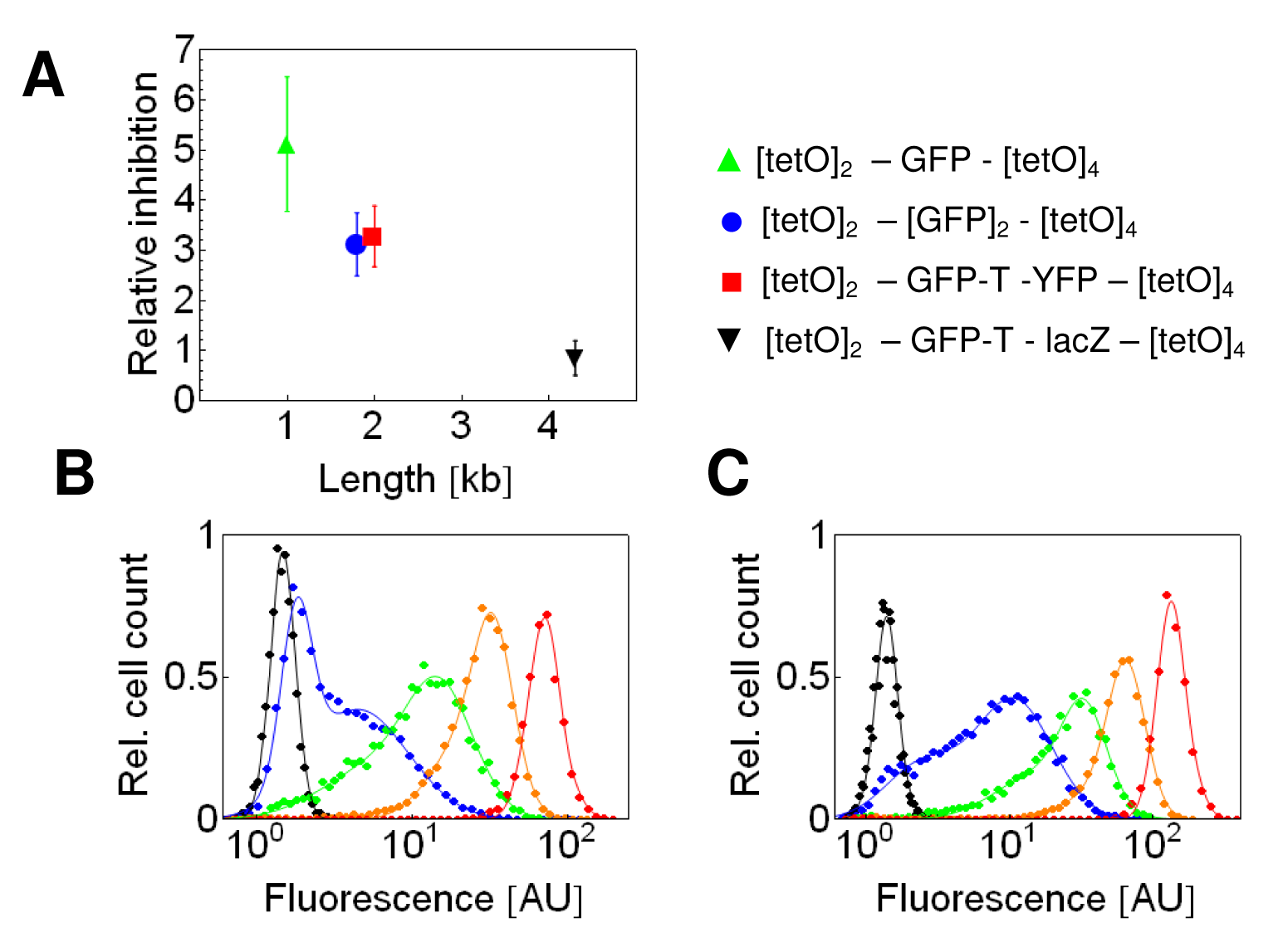

Supplement: Figure S10 — Collapse of bimodal expression as the distance between the recruitment sites for tetR-Sum1 is increased. (A) Sum1p was recruited to the dual recruitment constructs enclosing reporter genes of varying lengths (GFP, [GFP]2, GFP-T-YFP, GFP-T-lacZ integrated within the respective strains: YJKD-16, −3.4, −3.5, −3.6). The relative inhibition denotes the inhibition strength (see Materials and Methods) of the dual recruitment constructs normalized using the [tetO]2-GFP construct. The inhibition strength is the average value of the fold inhibition − 1 interpolated on the interval GA = [0.06, 0.6]. Error bars represent standard deviations calculated from three experiments. (B and C) Cellular fluorescence distributions due to the expression of the [tetO]2-[GFP]2-[tetO]4 (B) and [tetO]2-GFP-T-lacZ-[tetO]4 (C) constructs repressed by Sum1p. The cells were induced by 0, 4.1, 5.8, 16, and 200 nM estradiol, in the absence of doxycycline. No bimodal response was detected for the [tetO]2-GFP-T-lacZ-[tetO]4 construct. (0.36 MB TIF) [file pbio.1000332.s010.tif]

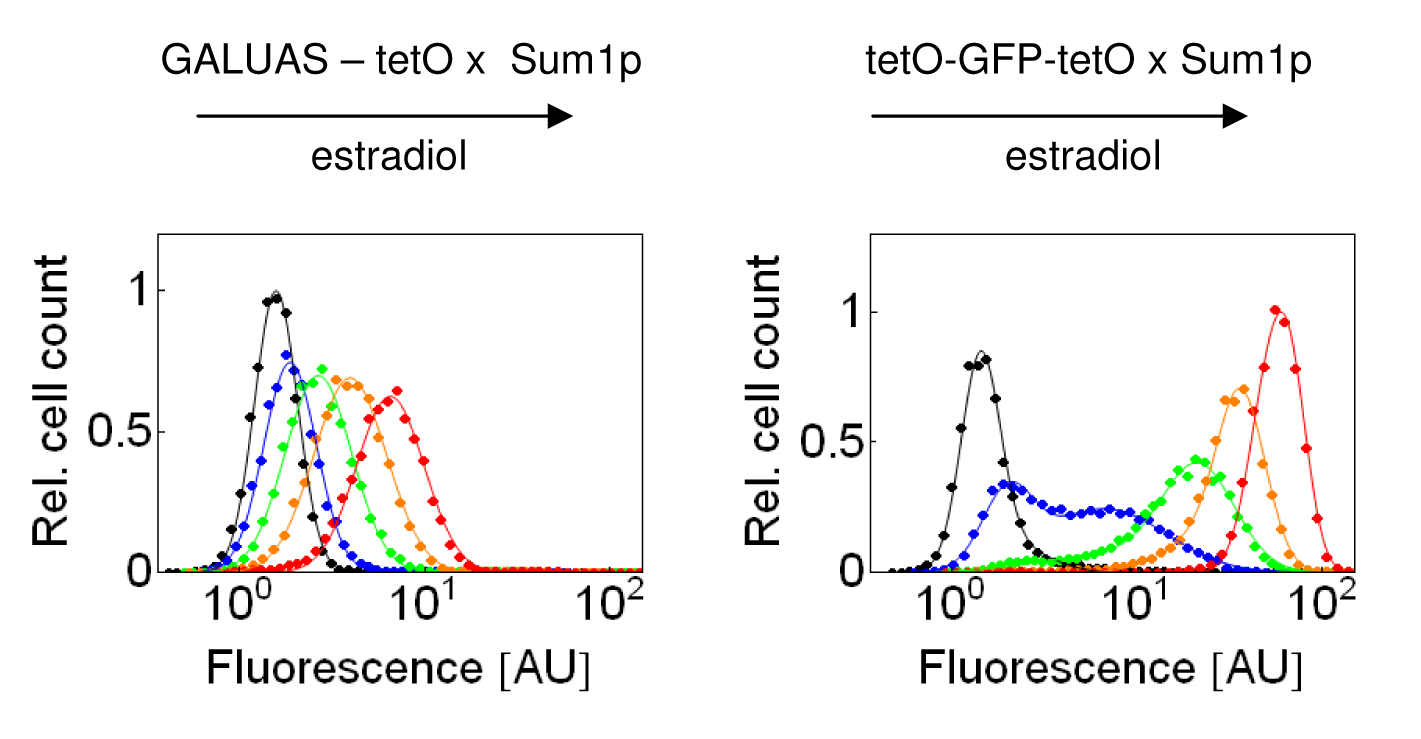

Supplement: Figure S11 — Cellular fluorescence distributions when expression is repressed by Sum1p. The cells (YJKD21.2.2 and YJK16) were induced by 3.75, 7.5, 15, 30, and 200 nM estradiol, in the absence of doxycycline. (0.24 MB TIF) [file pbio.1000332.s011.tif]

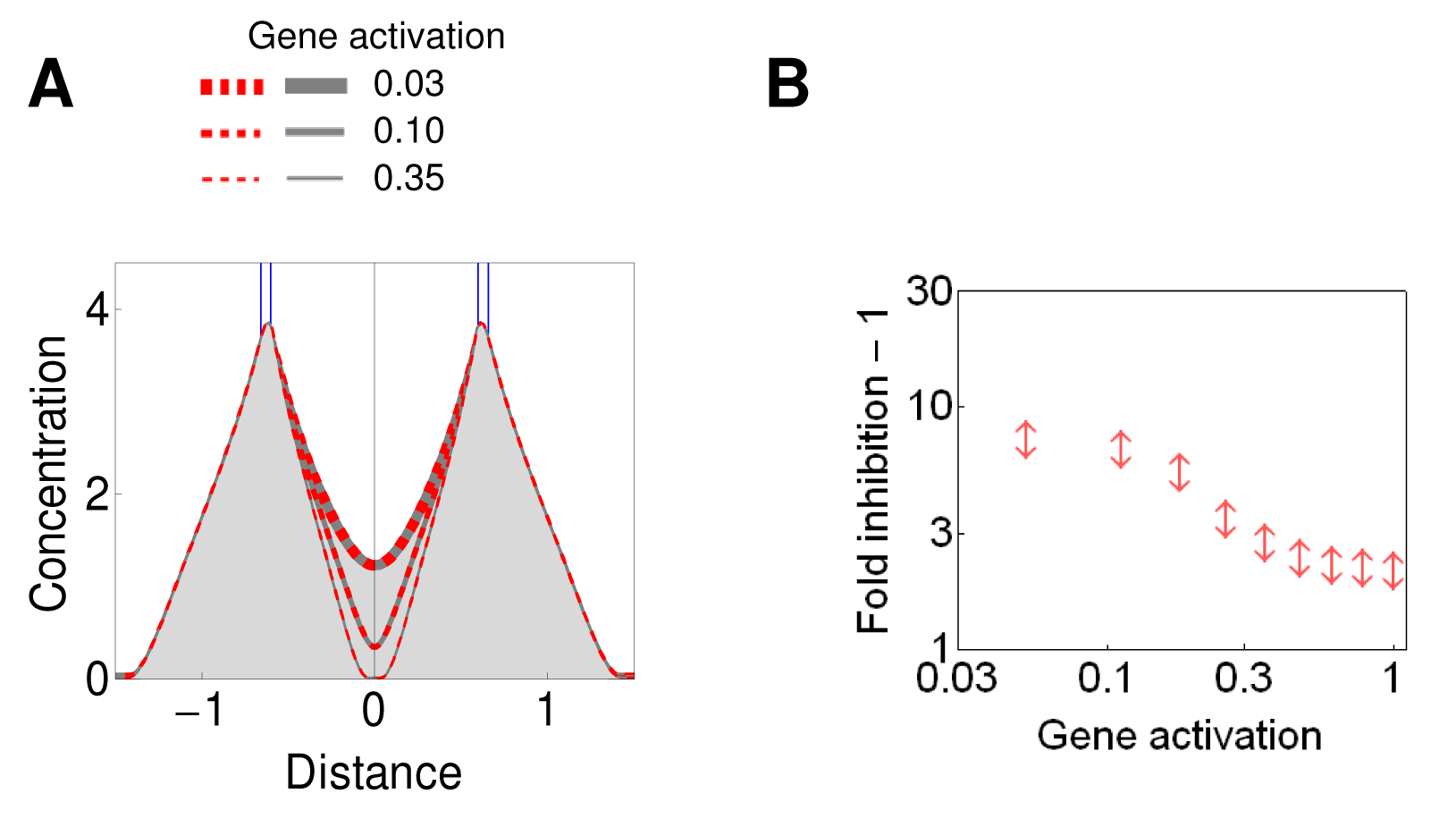

Supplement: Figure S12 — Monostable concentration profiles arise when cooperativity in the positive feedback loop is small. The Hill coefficient was reduced from 2 to n = 1.5. The following parameters were used for the simulations: s h = 6, L = 5, K = 5, b = 0.01, and kd = 1. The internucleation distance was 1.2 kb for the [O]2-Gene-[O]2 setting. (A) The red dashed and gray continuous lines represent the solutions initiated with low and high starting concentrations. The blue lines delimit the nucleation sites. When the two concentration profiles overlap red–gray dashed lines are visible. Monostable concentration profiles were obtained even at intermediate GA. (B) Inhibition of gene expression, expressed as fold inhibition − 1, was calculated from the values of the silencing concentration gradients. Even though there is no bistability at intermediate GA, a sigmoidal change in fold inhibition can be seen in this range. (0.25 MB TIF) [file pbio.1000332.s012.tif]

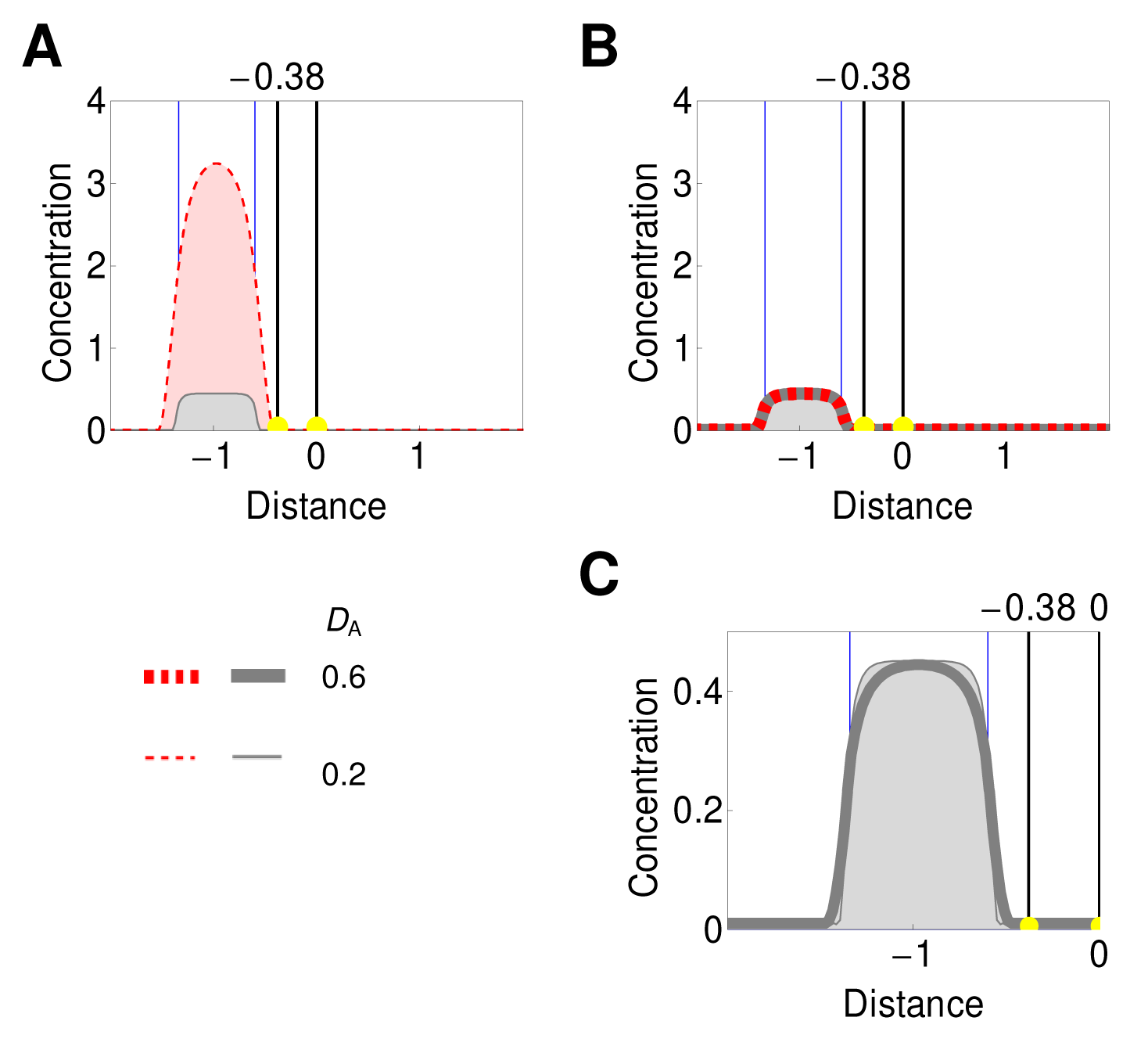

Supplement: Figure S13 — Bistable concentration profiles are confined to the proximity of the nucleating segment when diffusivity is low relative to the nucleation width. The following parameters were used for the simulations: s h = 0.3, L = 5, K = 7, b = 0.01, and k d = 1 for a [O]20-Gene setting. D A was set to the indicated values uniformly between the boundaries of the simulation. The blue lines delimit the nucleation segment, s w = 0.741 kb. The widening of the nucleation segment and reduction of the diffusivity renders the spatial aspect of the reaction–diffusion system less pronounced. Consequently, the behavior of the systems approximates that of a simple (nonspatial) positive feedback loop that generates bistability. The yellow dots denote the concentrations at −0.38 and 0 kb, which determine the level of GA. (A and B) The red dashed and gray continuous lines represent the solutions initiated with low and high starting concentrations with D A = 0.2 (A) and 0.6 (B). Bistable solution is obtained for lower diffusivity, D A = 0.2. It is evident that the silencing proteins do not propagate to long distances relative to the width of the nucleation segment and the concentrations of the silencing proteins at the gene regulatory region (yellow dots) are low even for the high-concentration profile. Thus, they have an effect on gene expression only in the vicinity of the nucleating segment. (C) The magnified version of the low-concentration profiles is displayed for D A = 0.2 (thin line) and 0.6 (thick line). It is evident that the concentration profile obtained for the lower diffusivity is more square-like. (0.29 MB TIF) [file pbio.1000332.s013.tif]
