# Supplementary material for: Spatial Epigenetic Control of Mono- and Bistable Gene Expression
Source: PLoS Biol. 2010 Mar 16;8(3):e1000332. doi: 10.1371/journal.pbio.1000332 (PMC2838748; doi:10.1371/journal.pbio.1000332)
Supplement: Table S1 — Constants used in the equations. (0.04 MB DOC) [file pbio.1000332.s014.doc]

### Table S1. Constants used in the equations

| **Constant** | **Constant Class** | **Description** | **Default Value** |
| --- | --- | --- | --- |
| *L* | Association | Maximum autocatalytic association rate of silencing proteins | 5 |
| *s*_h_ |  | Association rate of silencing proteins at the nucleation sites, whose width is denoted by *s_w_* | 6 |
| *b* |  | Basal nonspecific association rate of silencing proteins to the DNA | 0.01 |
| *k*_d_ | Dissociation | Dissociation rate of silencing proteins from the DNA | 1 |
| *D*_0_ | Diffusion | Basal diffusivity of silencing proteins along the DNA | 0.64 |
| *K* | Michaelis-like | It denotes the concentration at which the half maximal autocatalytic association rate is achieved. | 7 |
| *K_GA_* |  | It denotes the gene activation at which diffusivity equals *D_0_* / 2. | 0.77 |
| *n* | Hill coefficient | Describes the cooperativity of the binding of the silencing proteins to the DNA. *In vitro* measurements of the binding of Sir4 to Sir3 protein domains revealed a Hill coefficient of up to 3.5 (see reference King et al). Thus, it is realistic to assume a lumped Hill-coefficient of at least 2 for the overall binding process. | 2 |
| *s*_w_ | Geometry | Width of a nucleation segment. Corresponds to the total length of operators and inter-operator linker segments that build up one nucleation segment. For the given values, *s_w_*= (2*Num-1)*0.019, where Num denotes the number of operators. | Op *=*  0.019 kb linker = 0.019 kb |
| *Gene length* |  | It equals to the distance between the nucleation sites of the corresponding dual nucleation setting. The nucleation sources are positioned symmetrically with respect to the origin, with *s_w_* starting at the extremities of the gene, upstream (*s_wUP_*) and downstream (*s_wDOWN_*) respectively.  The source function *s(x)* is then a function of *s_h_*, *s_w_* and gene length, *l*:  *s_h_* , *x*  *A*  *s*(*x*) = {  0, *x*  *R* \ *A*  *A =* [ *-l* / 2 - *s_wUP_ , - l* / 2 ]  [*l* / 2 , *l* / 2 + *s_wDOWN_* ] | 1.2 kb |
