# Supplementary material for: Spatial Epigenetic Control of Mono- and Bistable Gene Expression
Source: PLoS Biol. 2010 Mar 16;8(3):e1000332. doi: 10.1371/journal.pbio.1000332 (PMC2838748; doi:10.1371/journal.pbio.1000332)
Supplement: Table S2 — Strains. (0.06 MB DOC) [file pbio.1000332.s015.doc]

### Table S2. Strains.

| Name | Regulators | Reporter gene | Origin |
| --- | --- | --- | --- |
| PRY-351 | GEV, tetR-Sir3p | *IYFR054C_TADH1_tetO2_PGAL1NR_GFP_TCYC1 _URA3* | pPR70→ BY4741 |
| PRY-355A | GEV, tetR-Sir3p | *IYFR054C_TADH1_tetO2_PGAL1NR_GFP_TADH1_tetO4_ TACT1_URA3* | pPR74→ BY4741 |
| PRY-509.3 | GEV, tetR-Sir3p | *IYFR054C_TADH1__tetO2_PGAL1NR_GFP_TCYC1_ tetO2_ TACT1_URA3* | pPR122→BY4741 |
| PRY-519.4 | GEV, tetR-Sir3p | *IYFR054C_TGAL7_PGAL1NR_GFP_TACT1_GALUAS_ TADH1_tetO4_ TACT1_URA3* | pPR128→ BY4741 |
| PRY-524.4 | GEV, tetR-Sir3p | *IYFR054C_TADH1_tetO2_PGAL1NR_GFP_TACT1_GALUAS_TADH1_tetO4_ TACT1_URA3* | pPR132→ BY4741 |
| PRY-527.5 | GEV, tetR-Sir3p | *IYFR054C_TGAL7_ PGAL1NR_GFP_TCYC1_ tetO2_ TACT1_URA3* | pPR131→ BY4741 |
| PRY-541.3 | GEV, tetR-Sir3p | *IYFR054ter_TetO1_PGAL1NR_GFP_TCYC1_tetO2_ TACT1_URA3* | pPR145→ BY4741 |
| PRY-544.1 | GEV, tetR-Sir3p | *IYFR054ter_I-silencer-PGAL1NR -GFP_TADH1_ tetO4-TACT1_URA3* | pPR151→ BY4741 |
| PRY-545.1 | GEV, tetR-Sum1p | *I_YFR054ter_ _I-silencer_PGAL1NR_GFP_TADH1_ tetO4_ TACT1_URA3* | pPR151→ BY4741 |
| PRY-548 | GEV, tetR-Sir3p | *IYFR054C_TGAL7_PGAL1NR_GFP_TCYC1_tetO1_ TACT1_URA3* | pMA05→ BY4741 |
| PRY-549 | GEV, tetR-Sir3p | *I_YFR054ter_ _TetO1_ PGAL1NR_GFP_TCYC1_ tetO1_ TACT1_URA3* | pMA04→ BY4741 |
| PRY-550 | GEV, tetR-Sir3p | *I_YFR054ter_ _TetO1_ PGAL1NR_GFP_TCYC1 _URA3* | pMA02→ BY4741 |
| YJK-14 | GEV, tetR-Sum1p | *I_YFR054C_ _TADH1_ tetO2_PGAL1NR_GFP_ TCYC1_URA3* | YSSH162.2 |
| YJK-15 | GEV, tetR-Sum1p | *I_YFR054C_ _TGAL7_ PGAL1NR_GFP_ TADH1_tetO4_T_ACT1_ _URA3* | YSSH167.3 |
| YJK-16 | GEV, tetR-Sum1p | *I_YFR054C_ _TADH1_ tetO2_PGAL1NR_GFP_ TADH1_tetO4_T_ACT1_ _URA3* | YSSH168.4 |
| YJKD-3.4 | GEV, tetR-Sum1p | *IYFR054C_TADH1_tetO2_PGAL1NR_GFP_GFP_TADH1_tetO4_ T_ACT1_ _URA3* | PRY500.5 |
| YJKD-3.5 | GEV, tetR-Sum1p | *I_YFR054C_ _TADH1_ tetO2_PGAL1NR_GFP_TACT1_YFP_TADH1_ tetO4_ T_ACT1_ _URA3* | YSSH175.7 |
| YJKD-3.6 | GEV, tetR-Sum1p | *I_YFR054C_ _TADH1_ tetO2_PGAL1NR_GFP_TACT1_lacZ_TADH1_ tetO4_ T_ACT1_ _URA3* | YSSH183.1 |
| YJKD-27 | GEV, tetR-Sum1p | *I_YFR054C_ _TGAL7_ PGAL1NR_mCherry_ TCYC1_tetO2_TACT1_ GFP_PGAL1NR_ TGAL7_URA3* | pJK26 → BY4741 |
| YJKD-33 | GEV, tetR-Sum1p | *I_YFR054C_ _TADH1_ tetO2_PGAL1NR_ mCherry_TCYC1_tetO2_TACT1_GFP_PGAL1NR_ TGAL7_URA3* | pJK27 → BY4741 |
| YJKD-21.2.2 | GEV, tetR-Sum1p | *I_YFR054C_ _TGAL7_ PGAL1tetO2_GFP_ TCYC1_URA3* | pJK30 → BY4741 |
| YJKD-19.1 | GEV, tetR-Sir3p | *IYFR054C_TGAL7_PGAL1NR_mCherry_TCYC1_tetO2_TACT1_ GFP_PGAL1NR_TGAL7_URA3* | pJK26 → BY4741 |
| YJKD20.2 | GEV, tetR-Sir3p | *I_YFR054C_ _TADH1_ tetO2_PGAL1NR_ mCherry_TCYC1_tetO2_TACT1_GFP_PGAL1NR_ TGAL7_URA3* | pJK27→ BY4741 |
| YJKD-21.2.1 | GEV, tetR-Sir3p | *IYFR054C_TGAL7_ PGAL1tetO2_GFP_ TCYC1_URA3* | pJK30→ BY4741 |
| YSSD-227.4 | GEV, tetR-Sir3p | *IYFR054C_TADH1_tetO7_PGAL1NR_GFP_TACT1_GALUAS_GFP_TCYC1 _URA3* | pPR61→BY4741 |
| DHS-43 | GEV, tetR-Sum1-1p | *I_YFR054C_ _TADH1_ tetO2_PGAL1NR_GFP_ TCYC1_URA3* | BY4741  sum1:  LEU2-SUM1-1 |
| DHS-44 | GEV, tetR-Sum1-1p | *I_YFR054C_ _TGAL7_ PGAL1NR_GFP_ TADH1_tetO4_T_ACT1_ _URA3* | BY4741  sum1:  LEU2-SUM1-1 |
| DHS-45 | GEV, tetR-Sum1-1p | *I_YFR054C_ _TADH1_ tetO2_PGAL1NR_GFP_ TADH1_tetO4_T_ACT1_ _URA3* | BY4741  sum1:  LEU2-SUM1-1 |
