# Supplementary material for: Spatial Epigenetic Control of Mono- and Bistable Gene Expression
Source: PLoS Biol. 2010 Mar 16;8(3):e1000332. doi: 10.1371/journal.pbio.1000332 (PMC2838748; doi:10.1371/journal.pbio.1000332)
Supplement: Table S3 — Plasmids. (0.05 MB DOC) [file pbio.1000332.s016.doc]

### Table S3. Plasmids

| **Plasmid Number** | **Description** | **Construction** |
| --- | --- | --- |
| pPR1 | pRS303::PMRP7-GEV-TACT1 | PMRP7-GEV Insert described in Gao & Pinkham, 2000: ApaI-PMRP7-SgsI-GEV-EcoRI-TACT1 -NotI |
| pPR13 | pRS306::*PRET2*(Int)-tetR-SIR3P-TACT1 | KpnI-PRET2-XbaI-tetR-HindIII-BamHI-SIR3P-SpeI-Tact1-NotI |
| pPR61 | pRS306::*YFR054C*(Int)-tetO7-PGAL1NR-GFP-TCYC1 | KpnI-YFR054C-SalI/XhoI-TADH1-tetO7-SphI-PGAL1NR-BamHI-GFP-EcoRI-TCYC1-NotI |
| pPR70 | pRS306::*YFR054C*(Int)- TADH1-tetO2 -PGAL1NR-GFP-TCYC1 | KpnI-YFR054C-SalI/XhoI-TADH1-tetO2-SphI-PGAL1NR-BamHI-GFP-EcoRI-TCYC1-NotI |
| pPR74 | pRS306::*YFR054C*(Int)- TADH1-tetO2 -PGAL1NR-GFP-tetO4 | KpnI-YFR054C-SalI/XhoI-TADH1-tetO2-SphI-PGAL1NR-BamHI-GFP-EcoRI-TADH1-tetO4-SphI-TACT1-NotI |
| pPR122 | pRS306::*YFR054C*(Int)- TADH1-tetO2 -PGAL1NR-GFP-tetO2 | KpnI-YFR054C-SalI/XhoI-TADH1-tetO2-SphI-PGAL1NR-BamHI-GFP-EcoRI-TCYC1-XbaI/SpeI-TetO2-SgsI-TACT1-NotI |
| pPR128 | pRS306::*YFR054C*(Int)-TGAL7-PGAL1NR-GFP-GALUAS-tetO4 | KpnI-YFR054C-SalI/XhoI-TGAL7-SphI-PGAL1NR-BamHI-GFP-SpeI-TACT1-BglII/ BamHI-GALUAS-EcoRI-TADH1-tetO4-SphI-TACT1-NotI |
| pPR131 | pRS306::*YFR054C*(Int)-TGAL7-PGAL1NR-GFP-tetO2 | KpnI-YFR054C-SalI/XhoI-TGAL7-SphI-PGAL1NR-BamHI-GFP-EcoRI-TCYC1-XbaI/SpeI-TetO2-SgsI-TACT1-NotI |
| pPR132 | pRS306::*YFR054C*(Int)- TADH1-tetO2 -PGAL1NR-GFP-GALUAS-tetO4 | KpnI-YFR054C-SalI/XhoI-TADH1-tetO2-SphI-PGAL1NR-BamHI-GFP-SpeI-TACT1-BglII/ BamHI-GALUAS-EcoRI-TADH1-tetO4-SphI-TACT1-NotI |
| pPR145 | pRS306::*YFR054C+ter*(Int)-tetO1-PGAL1NR-GFP- tetO2 | KpnI-YFR054C+ter-HindIII-tetO1-SgsI- PGAL1NR-BamHI-GFP-EcoRI-TCYC1-XbaI/SpeI-TetO2-SgsI-TACT1-NotI |
| pPR151 | pRS306::*YFR054C+ter*(Int)-I-silencer-PGAL1NR-GFP- tetO4 | KpnI-YFR054C+ter-HindIII-I-silencer-SgsI-PGAL1NR-BamHI-GFP-EcoRI-TADH1-tetO4-SphI-TACT1-NotI |
| pMA02 | pRS306::*YFR054C+ter*(Int)-tetO1-PGAL1NR-GFP-TCYC1 | KpnI-YFR054C+ter-HindIII-tetO1-SgsI- PGAL1NR-BamHI-GFP-EcoRI-TCYC1-NotI |
| pMA04 | pRS306::*YFR054C+ter*(Int)-tetO1-PGAL1NR-GFP- tetO1 | KpnI-YFR054C+ter-HindIII-tetO1-SgsI- PGAL1NR-BamHI-GFP-EcoRI-TCYC1-tetO1-TACT1-NotI |
| pMA05 | pRS306::*YFR054C*(Int)-TGAL7-PGAL1NR-GFP-tetO1 | KpnI-YFR054C-SalI/XhoI-TGAL7-SphI-PGAL1NR-BamHI-GFP-EcoRI-TCYC1-tetO1-TACT1-NotI |
| pJK22 | pRS306::PRET2(Int)-tetR-SUM1-TACT1 | KpnI-PRET2-XbaI-tetR-HindIII-BamHI-*SUM1*-SpeI-T_ACT1_-NotI |
| pJK26 | pRS306::YFR054C(Int)-TGAL7-PGAL1NR-mCherry-tetO2-GFP-PGAL1NR | KpnI-YFR054C-SalI/XhoI-T_GAL7_-SphI-P_GAL1NR_-BamHI-mCherry-EcoRI-T_CYC1_-XbaI/SpeI-TetO2-SgsI-T_ACT1_-SpeI-GFP-BamHI-P_GAL1NR_ -SphI-T_GAL7_-NotI |
| pJK27 | pRS306::YFR054C(Int)- TADH1-tetO2 -PGAL1NR-mCherry-tetO2-GFP-PGAL1NR | KpnI-YFR054C-SalI/XhoI-T_ADH1_-tetO2-SphI-P_GAL1NR_-BamHI-mCherry-EcoRI-TCYC1-XbaI/SpeI-TetO2-SgsI-TACT1-SpeI-GFP-BamHI-P_GAL1NR_ -SphI-T_GAL7_-NotI |
| pJK30 | pRS306::YFR054C(Int)-TGAL7-PGAL1tetO2-GFP | KpnI-YFR054C-SalI/XhoI-T_GAL7_-SphI-P_GAL1tetO2_ BamHI-GFP-EcoRI-T_CYC1_-NotI |
| pSS35 | pRS306::PRET2(Int)-tetR-SUM1-1-TACT1 | KpnI-PRET2-XbaI-tetR-HindIII-BamHI-SUM1-1-SpeI-TACT1-NotI |

**Description of sequences**

**GALUAS (four Gal4p binding sites)**

The DNA sequence corresponds to the -464 to -275 region of the GAL1 promoter.

ATATTGAAGTACGGATTAGAAGCCGCCGAGCGGGCGACAGCCCTCCGACGGAAGACTCTCCTCCGTGCGTCCTCGTCTTCACCGGTCGCGTTCCTGAAACGCAGATGTGCCTCGCGCCGCACTGCTCCGAACAATAAAGATTCTACAATACTAGCTTTTATGGTTATGAAGAGGAAAAATTGGCAGTAA

**PGAL1NR**

SphI-GALUAS-SalI-GAL1TATA-BamHI (Mig1 binding sites are deleted: NR-no repression)

GCATGCATATTGAAGTACGGATTAGAAGCCGCCGAGCGGGCGACAGCCCTCCGACGGAAGACTCTCCTCCGTGCGTCCTCGTCTTCACCGGTCGCGTTCCTGAAACGCAGATGTGCCTCGCGCCGCACTGCTCCGAACAATAAAGATTCTACAATACTAGCTTTTATGGTTATGAAGAGGAAAAATTGGCAGTAACCTGGCCCCACAAACCTTCAAATTAACGAATCAAATTAACAACCATAGGATGATAATGCGATTAGTTTTTTAGCCTTATTTCTGGGGTAATTAATCAGCGAAGCGATGATTTTTGATCTATTAACAGATATATAAATGGAAAAGCTGCATAACCACTTTAACTAATACTTTCAACATTTTCAGTTTGTATTACTTCTTATTCAAATGTCATAAAAGTATCAACAAAAAATTGTTAATATACCTCTATACTTTAACGTCAAGGAGAAAAAACTATAGGATCC

**PGAL1tetO2**

Is identical with the *GAL1* promoter with both Mig1 binding sites mutated into *tet* operators.

**PMRP7-GEV (Gal4DNA binding domain, Estradiol receptor, VP16 activator domain)**

Promoter Region of MRP7 (-904 to +1) and GEV of pGEVLeu2 (Gao & Pinkham, 2000) were ligated with SgsI and then cloned into pRS303 at the ApaI and EcoRI restriction sites.

**tetR**

Sequence between XbaI and HindIII of pCM217 that binds to *tet* operator.

**tetO** = *tet* operator

TCCCTATCAGTGATAGAGA

**tetO4**

tetO4 is a jumping product obtained from tetO7 during Polymerase Chain Reaction. A fifth operator within the PCR product contains a mutation.

**I-silencer**

The DNA sequence corresponds to coordinates 14575 to 14849 on Chromosome III.

AACTTACTTCAACATGAAAGCCCGACGTTTGCAATGATATCCTCTTTTTCACCCGATTATTTTCATTTATTAGTAAGTGGGGTTCTGGGTGAAAAAATCAAAACAAAAATATGAATTTGTAATGATTTTTATATTTTCGCCGGAGGTGCTGGAAATGGCAAACGAAAATACTATGACATAAAAGCTGGGCACACTATGTACGTTCTTTTTAATTTTTTATCAACATGAGAAAAATTCATGAACACTGTACCTTATATTATCTAATAAATGAAGTA

**ABBREVIATIONS**

tetO4 TADH1-tetO4

tetO7 TADH1-tetO7

PRET2 -710 to +12 of RET2

TADH1 ADH1 Terminator

TACT1 ACT1 Terminator

TGAL7 GAL7 Terminator

TCYC1 CYC1 Terminator

PMRP7 -904 to +1 of MRP7

YFR054C +1 to +413 of YFR054C

YFR054C+ter +1 to +987 of YFR054C
