# Supplementary material for: Spatial Epigenetic Control of Mono- and Bistable Gene Expression
Source: PLoS Biol. 2010 Mar 16;8(3):e1000332. doi: 10.1371/journal.pbio.1000332 (PMC2838748; doi:10.1371/journal.pbio.1000332)
Supplement: Text S1 — Supporting text and references. (0.05 MB DOC) [file pbio.1000332.s017.doc]

# Text S1 Supporting text and references

### Parameters affecting diffusion in the reaction – diffusion model

We simulated the binding of the activator by modulating the diffusion coefficient *D*_A_ locally, on the segment flanked by the nucleation sites. With increasing gene activation *D*_A_ was reduced.

The activator binding itself is not affected by silencing of chromatin [1,2]. The action of activators can be modeled using different assumptions concerning the mode of the antagonism between the activator and silencing proteins. Our model relies on non-competitive antagonism. Models displaying epigenetic bistability can be based on other forms of antagonism, as well [3]. It is interesting to note that histone acetylation can have different effects at different heterochromatic loci. For example, the Sas2p histone acetyl-transferase counteracts silencing at the HMR locus but enhances silencing at the HML locus [4,5]. Paradoxical effects due to special forms of antagonism have been observed for transcriptional interference, as well [6].

The 1D approximation is valid for most of the internal chromosomal segments. On the other hand, the telomeres display a higher degree of flexibility, and can form 3D structures, such as loops [7,8]. Modelling of the reaction and diffusion steps along shapes with radial symmetry can serve as a realistic approximation for loops. A single nucleation site within a circle, such as centromeric plasmids, may correspond to a dual nucleation setting in a 1D rod, since the silencing proteins would spread towards both ends of the gene within the circle.

### Numerical simulation of the partial differential equation

Equation 1 was solved numerically, fixing *c* = 0 at the boundaries of the simulated DNA segment, *c*(-*B*, *t*) = *c*(*B*, *t*) = 0, where *B* = 3 kb. The initial conditions are specified below. In bistable gene expression systems, transitions between the stable states are typically driven by stochastic fluctuations. To account for noise in the local concentration of silencing proteins, perturbations with two distinct concentration distributions of silencing proteins along the gene were applied at *t* = 0, and cycles were run until a sufficient convergence towards the steady-state, The nucleation of silencing proteins was required for stable long-term concentration profiles because in the absence of nucleation, the nonspecific association of silencing proteins was not sufficient to maintain concentration gradients initiated by a pulse (Figure S1). For the simulations, Matlab's *pdepe* partial differential equation solver was used, which employs the finite element method (FEM). 1 distance unit in this method corresponded to 0.01 kb. The discretization of space with the finite element method tallies with the scale of molecular movements along the DNA because Sir3p can associate with nucleosome-free DNA [9]; and it is reasonable to assume that a single Sir3p molecule covers approximately a 10 bp segment of DNA.

Some of the reactions may occur at a larger scale, at the level of nucleosomes; therefore we performed simulations with discretization at the scale of the nucleosomes. The simulations at both resolutions generated similar responses (compare Figures 1E, F and S2). The nucleosomes, themselves are not static, and they can move or dissociate from the DNA. Therefore, the nucleosome fluctuations can be averaged out, so that the finer discretization, such as used by the simulations with finite element method, can be applied even for the nucleosome based processes [3].

# Supporting references

1. Gao L, Gross DS (2008) Sir2 silences gene transcription by targeting the transition between RNA polymerase II initiation and elongation. Mol Cell Biol 28: 3979-3994.

2. Chen L, Widom J (2005) Mechanism of transcriptional silencing in yeast. Cell 120: 37-48.

3. Sedighi M, Sengupta AM (2007) Epigenetic chromatin silencing: bistability and front propagation. Phys Biol 4: 246-255.

4. Osada S, Kurita M, Nishikawa J, Nishihara T (2005) Chromatin assembly factor Asf1p-dependent occupancy of the SAS histone acetyltransferase complex at the silent mating-type locus HMLalpha. Nucleic Acids Res 33: 2742-2750.

5. van Welsem T, Frederiks F, Verzijlbergen KF, Faber AW, Nelson ZW, et al. (2008) Synthetic lethal screens identify gene silencing processes in yeast and implicate the acetylated amino terminus of Sir3 in recognition of the nucleosome core. Mol Cell Biol 28: 3861-3872.

6. Buetti-Dinh A, Ungricht R, Kelemen JZ, Shetty C, Ratna P, et al. (2009) Control and signal processing by transcriptional interference. Mol Syst Biol 5: 300.

7. Maillet L, Boscheron C, Gotta M, Marcand S, Gilson E, et al. (1996) Evidence for silencing compartments within the yeast nucleus: a role for telomere proximity and Sir protein concentration in silencer-mediated repression. Genes Dev 10: 1796-1811.

8. Zaman Z, Heid C, Ptashne M (2002) Telomere looping permits repression "at a distance" in yeast. Curr Biol 12: 930-933.

9. McBryant SJ, Krause C, Woodcock CL, Hansen JC (2008) The silent information regulator 3 protein, SIR3p, binds to chromatin fibers and assembles a hypercondensed chromatin architecture in the presence of salt. Mol Cell Biol 28: 3563-3572.
